# High-Resolution Transcriptome of Human Macrophages

**DOI:** 10.1371/journal.pone.0045466

**Published:** 2012-09-21

**Authors:** Marc Beyer, Michael R. Mallmann, Jia Xue, Andrea Staratschek-Jox, Daniela Vorholt, Wolfgang Krebs, Daniel Sommer, Jil Sander, Christina Mertens, Andrea Nino-Castro, Susanne V. Schmidt, Joachim L. Schultze

**Affiliations:** 1 Genomics and Immunoregulation, LIMES-Institute, University of Bonn, Bonn, Germany; 2 Department of Obstetrics and Gynecology, University of Bonn, Bonn, Germany; University of Freiburg, Germany

## Abstract

Macrophages are dynamic cells integrating signals from their microenvironment to develop specific functional responses. Although, microarray-based transcriptional profiling has established transcriptional reprogramming as an important mechanism for signal integration and cell function of macrophages, current knowledge on transcriptional regulation of human macrophages is far from complete. To discover novel marker genes, an area of great need particularly in human macrophage biology but also to generate a much more thorough transcriptome of human M1- and M1-like macrophages, we performed RNA sequencing (RNA-seq) of human macrophages. Using this approach we can now provide a high-resolution transcriptome profile of human macrophages under classical (M1-like) and alternative (M2-like) polarization conditions and demonstrate a dynamic range exceeding observations obtained by previous technologies, resulting in a more comprehensive understanding of the transcriptome of human macrophages. Using this approach, we identify important gene clusters so far not appreciated by standard microarray techniques. In addition, we were able to detect differential promoter usage, alternative transcription start sites, and different coding sequences for 57 gene loci in human macrophages. Moreover, this approach led to the identification of novel M1-associated (CD120b, TLR2, SLAMF7) as well as M2-associated (CD1a, CD1b, CD93, CD226) cell surface markers. Taken together, these data support that high-resolution transcriptome profiling of human macrophages by RNA-seq leads to a better understanding of macrophage function and will form the basis for a better characterization of macrophages in human health and disease.

## Introduction

Macrophages represent resident phagocytic cells in the tissue and are involved in tissue homeostasis and induction of inflammatory reaction towards pathogens by use of their broad range of pattern-recognition receptors [1]. In context of the respective immune response, macrophages are polarized to specific functional properties, often referred to as M1-like and M2-like phenotype. Human monocytes can be differentiated towards macrophages using either M-CSF or GM-CSF already resulting in differences in gene expression [2]. Classically polarized M1-like macrophages can be induced by IFN-γ alone or together with LPS or TNF-α using M-CSF or GM-CSF [3]. M1-like macrophages are effector cells of classical inflammatory immune responses exerting an IL-12^high^, IL-23^high^ and IL-10^low^ phenotype with secretion of inflammatory cytokines IL-1β, IL-6 and TNF-α. They display a phenotype characterized by the expression of CD86, CD64, and CD16 [4], [5]. In contrast, macrophages that are activated by other mechanisms than IFN-γ/LPS/TNF-α are grouped in the alternatively activated M2-like macrophage subset. Non-classically activated macrophages can be induced by cytokines including IL-4 and IL-13, but other stimuli have been described as well [4], [5]. These cells share an IL-12^low^ and IL-23^low^ phenotype and express CD23. Over the last decade, phenotypic adaptations of macrophages to environmental stimuli have been linked to radical changes in transcriptional regulation mainly by applying microarray-based gene expression profiling [3], [6]–[9]. In fact, a large amount of data covering transcriptional reprogramming of macrophages has been accumulated, albeit not always systematic [3], [6]–[11]. However, molecular mechanisms controlling transcriptional reprogramming in macrophages are far from understood and it has been suggested that integrative analyses of epigenomic and transcriptomic data will be required to better understand how macrophages integrate the information they receive from their respective microenvironment [12], enabling the identification of specific transcription factor combinations being responsible for cellular macrophage programs.

**Figure 1:**
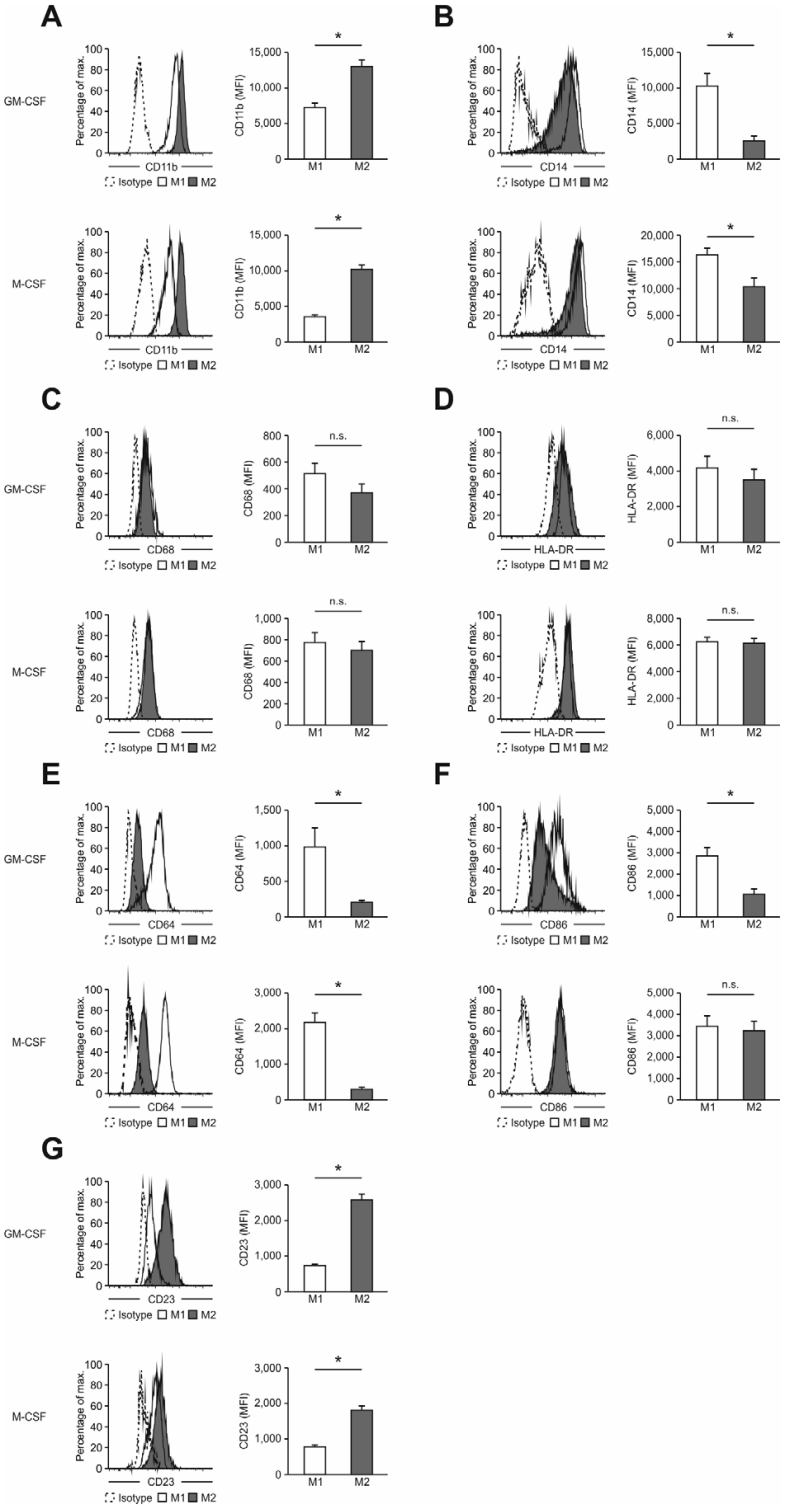
Phenotypic characterization of human M1- and M2-like macrophages derived from CD14^+^ peripheral blood monocytes. Expression of typical macrophage lineage markers was determined by flow cytometry (left) of M1- and M2-like macrophages generated in the presence of GM-CSF (upper panel) or M-CSF (lower panel) with quantification shown in the graph at the right. Expression of (A) CD11b, (B) CD14, (C) CD68, (D) HLA-DR, (E) CD64, (F) CD86, and (G) CD23, respectively. Isotype controls are depicted as dotted lines. *P<0.05 (Student’s t-test). Numbers in plots indicate mean fluorescence intensity. Data are representative of nine independent experiments (A,B,D,E,F,G; mean and s.e.m.) or eight independent experiments (C; mean and s.e.m.), each with cells derived from a different donor.

The introduction of RNA sequencing (RNA-seq) to interrogate whole transcriptomes has challenged previously established gene expression profiling studies [13]–[15]. Advantages assigned to RNA-seq over microarray analysis include increases in transcript quantity and quality, improved detection of alternative splicing events and gene fusion transcripts, and a larger dynamic range of detection [13]–[15].

**Figure 2:**
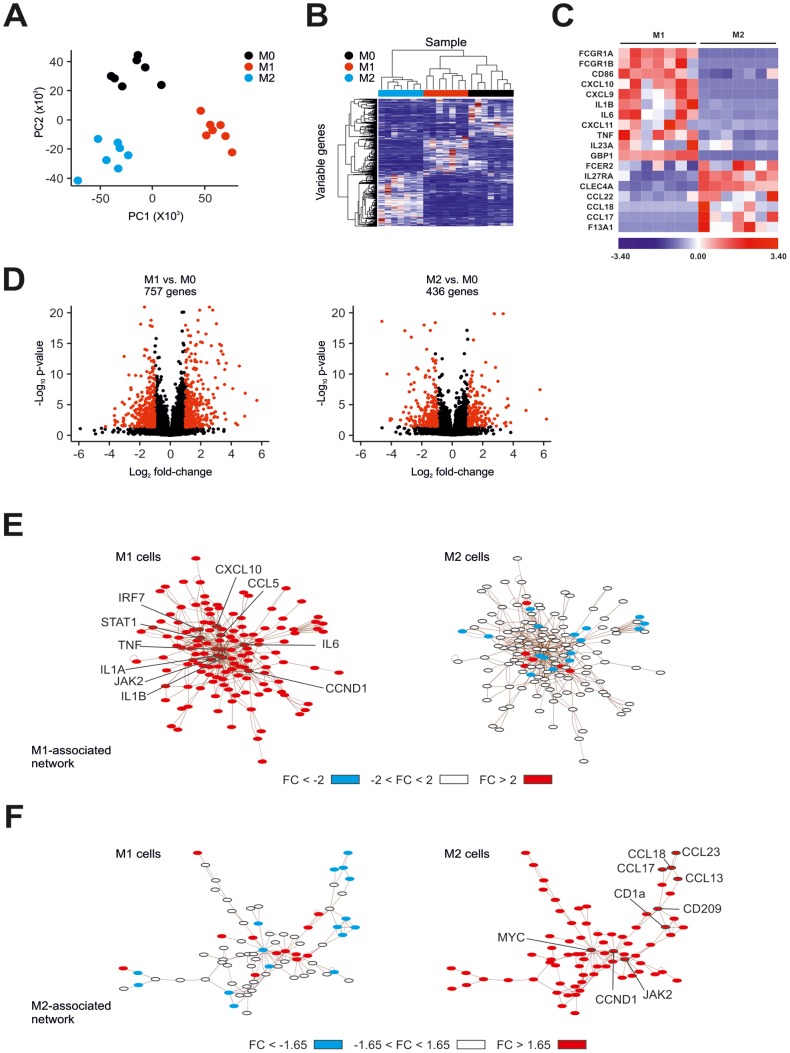
Microarray-based RNA fingerprinting of human M1- and M2-like macrophages. (A) Principle component analysis of human unpolarized (M0) and polarized (M1, M2) macrophages. (B) Unsupervised hierarchical clustering of human M0, M1-, and M2-like macrophages. (C) Visualization of known markers for human M1- and M2-like macrophages as a heatmap. Data were z-score normalized. (D) Volcano plots showing fold-change and p-value for the comparisons of M1-like versus M0 (left) and M2-like versus M0 macrophages (right). Differentially expressed genes (FC ≥2, p-value <0.05 with FDR, diff >100) are depicted in red. (E) Left: network of genes highly expressed in M1-like macrophages (fold-change >2.0) in comparison to M0 macrophages identified by microarray analysis. Right: data for the comparison of M2-like versus M0 macrophages were loaded into the M1-network. (F) Right: network of genes highly expressed in M2-like macrophages (fold-change >1.65) in comparison to M0 macrophages identified by microarray analysis. Left: data for the comparison of M1-like versus M0 macrophages were loaded into the M2-network. All networks were generated using EGAN.

To better understand polarization and integration of environmental signals by macrophages and to identify more specific markers for different functional states, high-resolution transcriptome data have been asked for [16]. Using M1 and M2 polarization as models we applied RNA-seq and compared the information content with data derived by microarray analysis. We provide new insights into human macrophage biology and determine several new markers associated with classical and alternative macrophage polarization in humans.

## Materials and Methods

### Ethics Statement

Buffy coats from healthy donors were obtained following protocols accepted by the institutional review board at the University of Bonn (local ethics vote no. 045/09). Informed written consent was provided for each specimen according to the Declaration of Helsinki.

### Cell Isolation from Healthy Blood Donors

Peripheral blood mononuclear cells (PBMC) were obtained by Pancoll (PAN-Biotech, Aidenbach, Germany) density centrifugation from buffy coats from healthy donors. CD14^+^ monocytes were isolated from PBMC using CD14-specific MACS beads (Miltenyi Biotec) according to the manufacturers protocol (routinely >95% purity, Figure S1A).

### Generation of Macrophages

CD14^+^ monocytes were cultured in 6-well plates in RPMI1640 medium containing 10% FCS and differentiated into immature macrophages using GM-CSF (500 U/ml) or M-CSF (100 U/ml) for 3 days (Figure S1B). Growth-factor containing medium was exchanged on day 3 and cells were polarized for 3 days with the following stimuli: IFN-γ (200 U/ml), TNF-α (800 U/ml), ultrapure LPS (LPSu, 10 µg/ml), IL-4 (1,000 U/ml), IL-13 (100 U/ml), or combinations thereof (all from Immunotools, Friesoythe, Germany, Figure S1C).

**Table 1:** RNA-seq.

	M1		M2	
	reads (x10^6^)	percentage (%)	reads (x10^6^)	percentage (%)
Total	15.0±2.8		20.4±9.2	
Exons	11.8±2.2	78.4±1.1	16.1±7.4	79.4±2.0
Exon-Intron boundaries	0.4±0.1	2.5±0.1	0.5±0.2	2.4±0.2
Introns	2.1±0.5	14.1±1.0	2.7±1.3	13.2±1.6
Intergenic regions	0.8±0.1	5.0±0.2	1.0±0.5	5.0±0.3
Average coverage	37.5±7.0		51.0±23.0	

**Figure 3:**
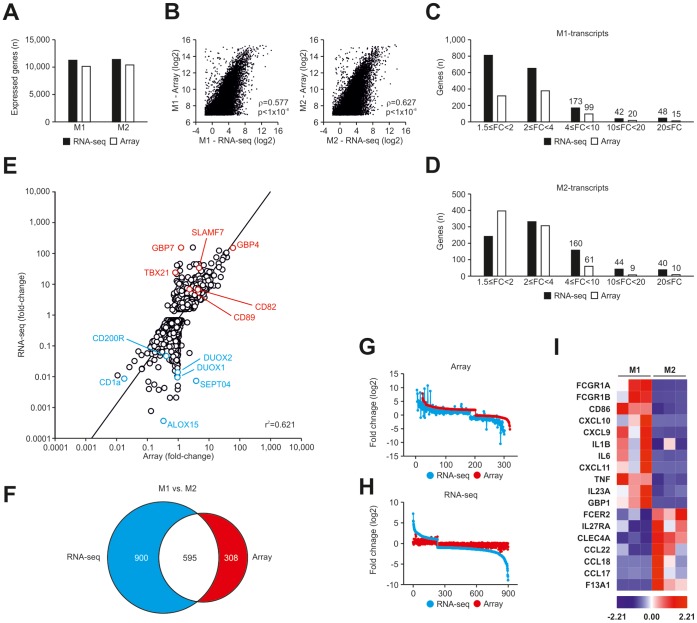
Comparison of RNA-seq and microarray analysis. (A) Number of genes expressed in human M1- (left) and M2-like macrophages (right) as detected using RNA-seq (black) and microarray analysis (white). (B) Correlation (Spearman) of mean expression values of M1- (left) and M2-like macrophages (right) using RNA-seq and microarray analysis. (C–D) Comparison of differentially expressed genes detected using RNA-seq or microarray analysis (p<0.05). Differentially expressed genes as assessed by RNA-seq (black) or microarray analysis (white) were divided into groups by their relative expression in (C) M1 versus M2 or (D) M2 versus M1. (E) Gene expression in M1- versus M2-like macrophages as fold change versus fold change plot comparing microarray analysis with RNA-seq using all Refseq genes differentially expressed in RNA-seq. (F) Venn-diagram of differentially expressed genes between M1- and M2-like macrophages (M1 vs. M2) in RNA-seq (blue) and microarray analysis (red), (FC ≥2, p-value <0.05 with FDR, diff >100 for microarray data). Fold-change-rank plots of genes detected as differentially expressed between M1- and M2-like macrophages (G) by microarray analysis (red) with overlay of values obtained by RNA-seq (blue) or (H) by RNA-seq (blue) with overlay of values obtained by microarray analysis (red). (I) Visualization of known markers for human M1- (left) and M2-like macrophages (right) from Fig. 2C as a heatmap using RNA-seq. Data were z-score normalized.

### Monoclonal Antibodies and Flow Cytometry

Cells were stained after FcR blockade incubating cells in PBS with 20% FCS for 10 minutes at 4°C using the following monoclonal antibodies (all from Becton Dickinson (BD), BioLegend, or eBioscience): FITC-labeled CD1b, CD23, CD93, CD226, CD82, anti-HLA-DR, anti-TLR2; PE-labeled CD64, CD68, CD120b,CD85j, CD85a, CD89, CD200R, anti-SLAMF7; PE-Cy5-labeled CD1a; PerCP-Cy5.5-labeled CD209; APC-labeled CD86, CD85h; Pacific Blue-labeled CD11b; and APC-Cy7-labeled CD14 with matched isotype antibodies as controls. Intracellular staining of CD68 was performed using the BD Cytofix/Cytoperm kit (BD). Data were acquired on a LSR II (BD) and analyzed using FlowJo software (Tree Star).

**Figure 4:**
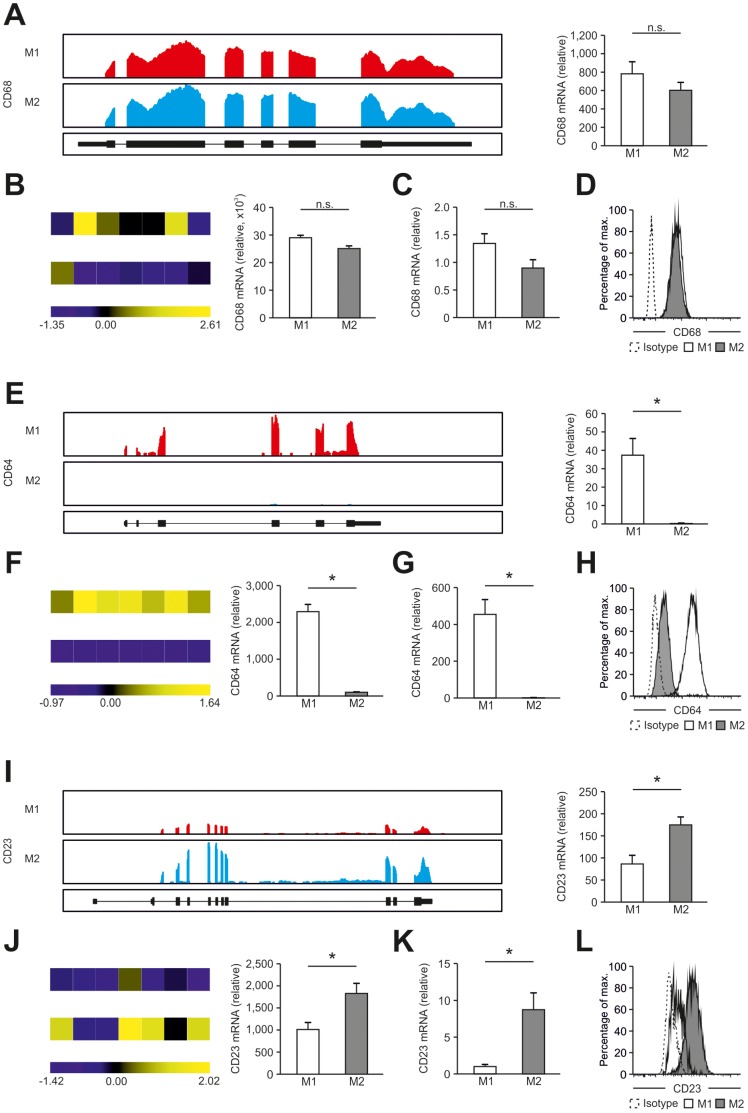
Correlation of RNA-seq, microarray, qPCR, and flow cytometric analysis. (A–D) CD68, (E–H) CD64, and (I–L) CD23 expression in human M1- and M2-like macrophages. (A, E, I) Left, representative images of sequencing reads across the genomic loci of genes expressed in human macrophages. Pictures taken from the Integrative Genomics Viewer (IGV). The height of bars represents the relative accumulated number of 100-bp reads spanning a particular sequence. Gene maps (bottom portion of each panel, oriented 5′-3′ direction) are represented by thick (exons) and thin (introns) lines. Right, RPKM values by RNA-seq in M1- and M2-like macrophages. (B, F, J) Left, heatmaps presenting microarray results from M1- and M2-like macrophages from seven donors. Data were z-score normalized. Right, relative mRNA expression. (C, G, K) Relative mRNA expression by qPCR in M1- and M2-like macrophages. (D, H, L) Protein expression was determined by flow cytometry in human M1- and M2-like macrophages. Data are representative of three experiments (RNA-seq, mean and s.e.m.), seven experiments (microarray, mean and s.e.m.), at least seven experiments (qPCR; mean and s.e.m.), and nine experiments (flow cytometry), each with cells derived from a different donor. Isotype controls are depicted as dotted lines. *P<0.05 (Student’s t-test).

### RNA Isolation

5×10^6^–2×10^7^ macrophages were harvested, subsequently lysed in TRIZOL (Invitrogen) and total RNA was extracted according to the manufactures’ protocol. The precipitated RNA was solved in RNAse free water. The quality of the RNA was assessed by measuring the ratio of absorbance at 260 nm and 280 nm using a Nanodrop 2000 Spectrometer (Thermo Scientific) as well as by visualization the integrity of the 28S and 18S band on an agarose gel.

**Figure 5:**
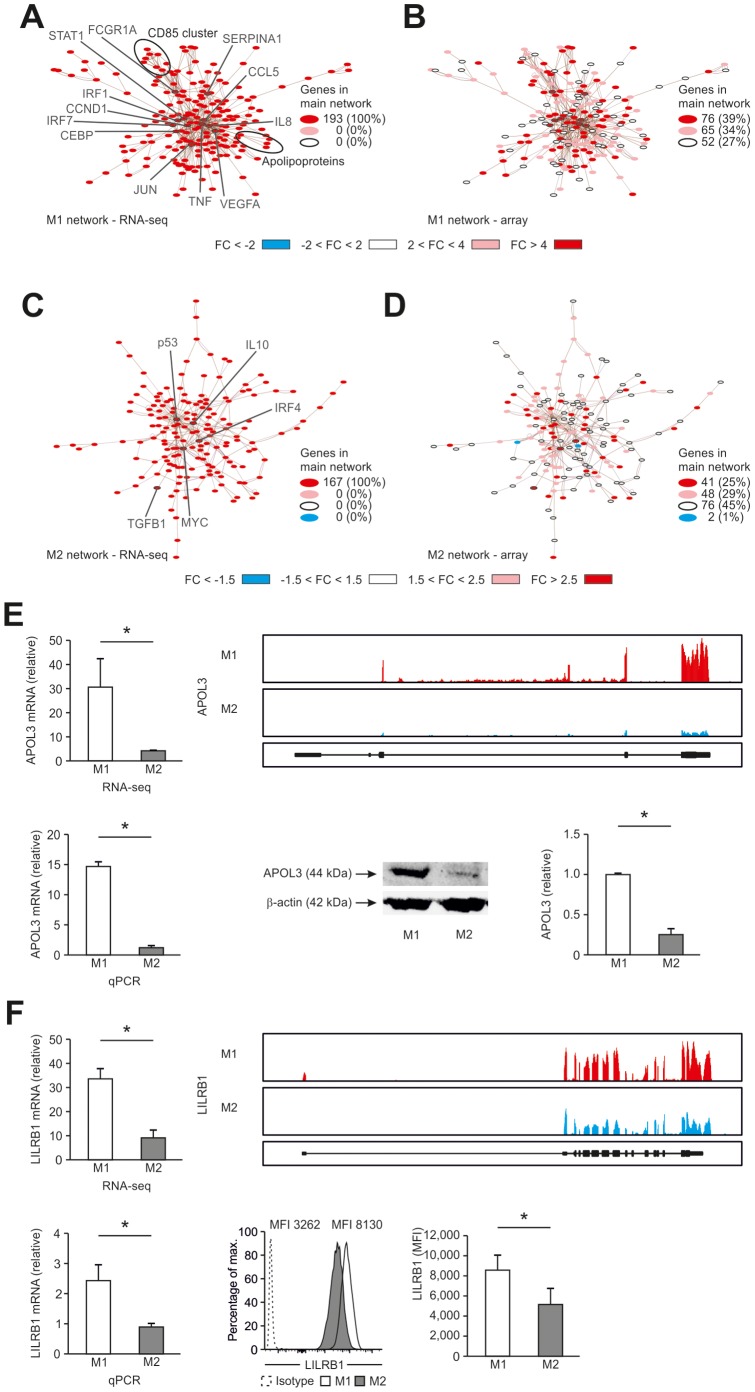
Network analysis of RNA-seq data. (A) Network of genes highly expressed in M1-like macrophages (fold-change >4.0) identified by RNA-seq. (B) Data generated by microarray analysis were loaded into the M1-network established using RNA-seq. (C) Network of genes highly expressed in M2-like macrophages (fold-change >2.5) identified by RNA-seq. (D) Data generated by microarray analysis were loaded into the M2-network established using RNA-seq. All networks were generated using EGAN. (E) APOL3 and (F) LILRB1 expression in human M1- and M2-like macrophages. Far left, relative expression as determined by RNA-seq. Left, representative images of sequencing reads across genes expressed in human macrophages as described in Fig. 4. Right, relative mRNA expression by qPCR in M1- and M2-like macrophages. Far right, protein data as determined by immunoblotting, respective flow cytometry. Data are representative of three experiments (RNA-seq, qPCR, and immunoblotting resp. flow cytometry; mean and s.e.m.) each with cells derived from a different donor. Isotype controls are depicted as dotted lines. *P<0.05 (Student’s t-test).

### Quantitative PCR Conditions and Primer Sequences

500 ng RNA was reverse transcribed using the Transcriptor First Strand cDNA Synthesis Kit (Roche Diagnostics). qPCR was performed using the LightCyclerTaqman master kit with GAPDH as reference on a LightCycler 480 II (Roche). GAPDH was chosen as reference as both microarray analysis as well as RNA-seq did not show statistically significant differences in gene expression for GAPDH in the conditions assessed. Primer sequences and assay conditions were determined using the Universal Probe Library Assay Design Center (Roche). qPCR primer sequences are summarized in Table S1.

Isoform specific PCR to identify alternative splicing events were performed using the Maxima SYBR Green/Fluorescein qPCR Master Mix (Fermentas). The relative enrichment of each isoform relative to GAPDH was calculated using the 2^−ΔΔCT^ method. Primer sequences and assay conditions were determined using Beacon Designer 7. qPCR primer sequences are listed in Table S2.

**Figure 6:**
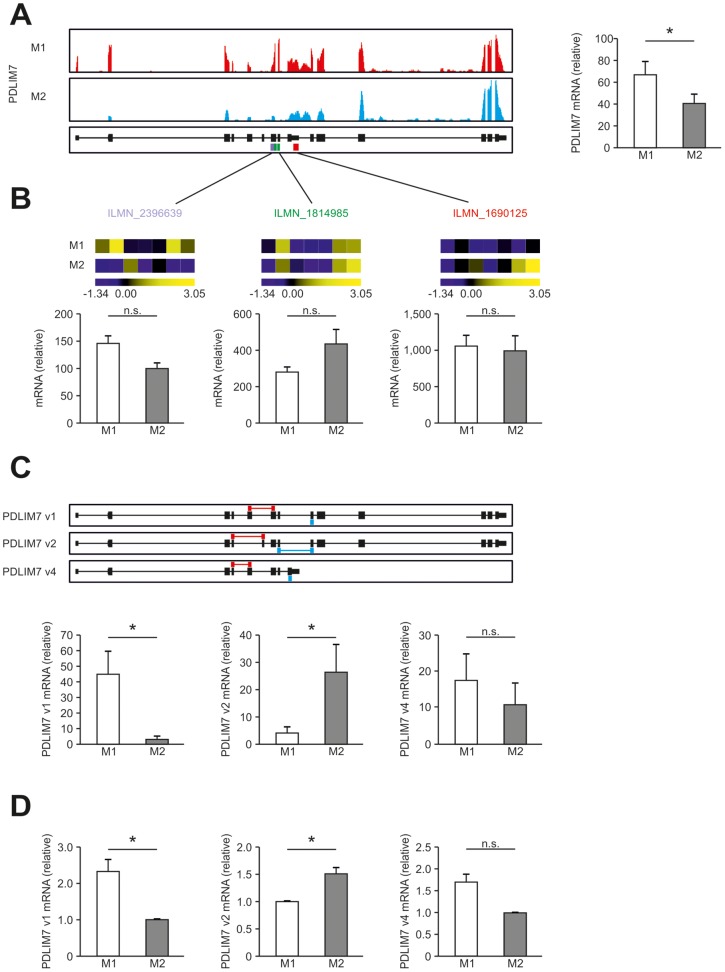
Detection of alternative splicing in human macrophages. (A) Summarized expression of all PDLIM7 transcripts in human M1- and M2-like macrophages. Left, representative images of sequencing reads across genes expressed in human macrophages as described in Fig. 4. Right, RPKM values for PDLIM7 by RNA-seq in M1- and M2-like macrophages. (B) Expression of PDLIM7 as determined by microarray analysis using 3 different probes recognizing different parts of the PDLIM7 transcripts as depicted in (A). (C) Upper panel: representation of the 3 different mRNA transcripts from Refseq. Lower panel: abundance of the different transcripts as determined using Cuffdiff. (D) qPCR for the 3 different mRNA transcripts from Refseq in human M1- and M2-like macrophages. Splice variant specific primers depicted in red and blue. Data are representative of three experiments (RNA-seq), seven experiments (microarray analysis) or at least ten experiments (qPCR; mean and s.e.m.), each with cells derived from a different donor. *P<0.05 (Student’s t-test).

**Figure 7:**
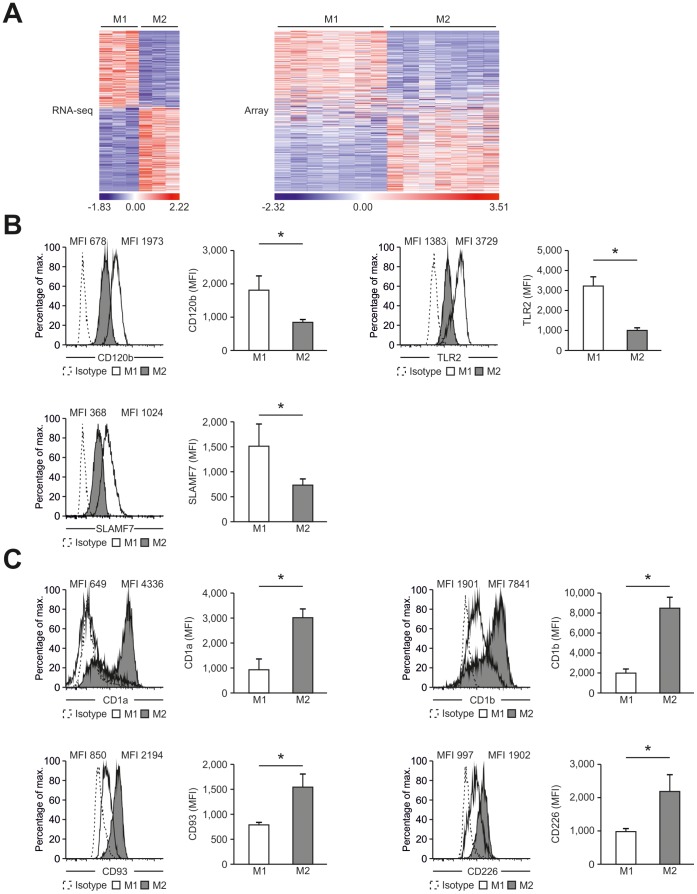
Identification of new macrophage polarization markers based on combined transcriptome analysis. (A) Differentially expressed genes between M1- and M2-like macrophages of the human surfaceome were visualized as heatmaps for RNA-seq (left) and microarray analysis (right). Data were z-score normalized. (B–C) Expression of novel macrophage markers was determined by flow cytometry (left) of M1- and M2-like macrophages generated in the presence of GM-CSF with quantification shown in the graph at the right. Expression of (B) CD120b, TLR2, and SLAM7 as well as (C) CD1a, CD1b, CD93, and CD226. Isotype controls are depicted as dotted lines. *P<0.05 (Student’s t-test). Numbers in plots indicate mean fluorescence intensity. Data are representative of nine independent experiments (B,C; mean and s.e.m.) each with cells derived from a different donor.

### Microarray-based Transcriptional Profiling and Bioinformatic Analysis of Microarray Data

Prior to array based gene expression profiling total RNA was further purified using the MinElute Reaction Cleanup Kit (Qiagen). Biotin labeled cRNA was generated using the TargetAmp Nano-g Biotin-cRNA Labeling Kit for the Illumina System (Epicentre). Biotin labeled cRNA (1.5 µg) was hybridized to Human HT-12V3 Beadchips (Illumina) and scanned on an Illumina HiScanSQ system. Raw intensity data were processed and exported with BeadStudio 3.1.1.0 (Illumina). Subsequent analyses were performed using the R programming language [17] with the Bioconductor software packages [18]. Quality of the array data was assessed using pairwise scatterplots whereby the correlation coefficient should account to r^2^≥0.95. Moreover the present call rate was calculated and only samples with at least 30% present calls were included in further studies. Having passed the quality control check points, data was normalized using quantile normalization implemented in the “limma” package of Bioconductor. [18]. To remove non-informative genes, the data was filtered using the coefficient of variation. Hence, only genes with a coefficient of variation of at least 0.5 were kept for further analysis. From the resulting data sets we extracted a list of genes with a significant different expression in macrophage subtypes (fold-change ≥2.0, p-value <0.05 Student’s *t*-test with Benjamini & Hochberg false-discovery rate correction). Variable genes were plotted as heatmaps with hierarchical clustering using the correlation coefficient as a distance measure for the samples and the average of each cluster for cluster formation of the genes using the “amap” package. Expression values are visualized with colors ranging from red (high expression) over white (indermediate expression) to blue (low expression). Principal component analysis (PCA) was performed using the “pcurve” package in R [19]. When visualizing PCA results, the first 2 principal components (coordinates) are shown z-transformed. Microarray data can be accessed under GSE35449.

### RNA-seq and Data Analysis

Sequencing and analysis were performed individually on M1-like and M2-like macrophages from 3 independent donors. Total RNA was converted into libraries of double stranded cDNA molecules as a template for high throughput sequencing using the Illumina CBot station and HiScanSQ following the manufacturer’s recommendations using the Illumina TruSeq RNA Sample Preparation Kit. Shortly, mRNA was purified from 5–10 µg of total RNA using poly-T oligo-attached magnetic beads. Fragmentation was carried out using divalent cations under elevated temperature in Illumina proprietary fragmentation buffer. First strand cDNA was synthesized using random oligonucleotides and SuperScript II. Second strand cDNA synthesis was subsequently performed using DNA Polymerase I and RNase H. Remaining overhangs were converted into blunt ends via exonuclease/polymerase activities and enzymes were removed. After adenylation of 3′ ends of DNA fragments, Illumina PE adapter oligonucleotides were ligated to prepare for hybridization. In order to select cDNA fragments of preferentially 200 bp in length the library fragments were separated on a 2% (w/v) agarose gel. The corresponding gel-fraction for each library was excised and purified using the QIAquick gel extraction kit (Qiagen). DNA fragments with ligated adapter molecules were selectively enriched using Illumina PCR primer PE1.0 and PE2.0 in a 15 cycle PCR reaction. Products were purified (QIAquick PCR purification kit) and quantified using the Agilent high sensitivity DNA assay on a Bioanalyzer 2100 system (Agilent). After cluster generation, 100 bp paired-end reads were generated and analyzed using CASAVA 1.8. Alignment to the human reference genome hg19 from UCSC was performed stepwise. First, all reads passing the chastity filter were aligned to the reference genome. Next, reads were aligned to the RNA reference transcriptome. Based on these alignments the numbers of reads aligning to intragenic regions, or intergenic regions, respectively, were calculated. In addition the numbers of reads mapping to exonic and intronic regions as well as to splice sites were calculated based on the UCSC annotation file [20]. Reads per kilobase of exon model per million mapped reads (RPKM) values for Refseq genes were established using CASAVA 1.8. In order to identify reads spanning altered splicing events or gene fusion breakpoints we also analyzed reads using TopHat and Bowtie. Results were further processed using Cufflinks and Cuffdiff [21]–[24]. RNA-seq data can be accessed under GSE36952.

### A Priori Information-based Network Analysis Using EGAN Software

To visualize connectivity between genes in high-throughput datasets contextual network graphs were generated based on a priori knowledge stored in literature, pathway, interaction, or annotation term databases by EGAN (exploratory gene association network) [25]. To visualize the transcriptional regulation of genes enriched in M1 respectively M2, array data were used and fold change differences calculated using unpolarized macrophages as comparison. Genes with a FC >2 for M1 and FC >1.65 for M2 were visualized; represented is the major network. Using the network topology established for M1-like macrophages the expression values for M2-like macrophages were plotted and vice versa. For comparison of network components and density between RNA-seq and array data, the network was first visualized for the RNA-seq data (FC >4 for M1 and FC >2.5 for M2). Keeping the network topology, genes were marked according to their fold change when visualizing the array-based network. Graphs for genes enriched in M1 respectively in M2 were generated independently.

### Immunoblot Analysis

Cell lysates from human macrophages were prepared as previously described [26] followed by immunoblotting with APOL1 or APOL3 antibodies (Abcam) as well as human β-actin (C4; Millipore) as loading control.

### Statistical Analysis

For flow cytometry or qPCR data comparisons between groups were performed using the appropriate paired or unpaired Student’s *t*-test after testing for equal variance or normal distribution of the data, respectively. P<0.05 was defined as statistically significant. The relationship between fold-changes in gene expression in RNA-seq and microarray analysis was investigated using linear regression. All statistical analyses were performed using the SPSS 19.0 statistical software package.

## Results

### Generation of Human M1- and M2-like Macrophages as a Model System

To establish a high-resolution transcriptome of human macrophages as a result of specific polarization signals, we used classical (M1-like) and alternative (M2-like) polarization of human macrophages as a model system. Since both M-CSF and GM-CSF have been described to differentiate macrophages from blood-derived CD14^+^ monocytes with distinct gene expression profiles [2], we first compared the two different stimuli in respect to macrophage polarization and used expression of well-known macrophage markers as the initial readout [3], [27]. For classical polarization we primarily used IFN-γ as the model stimulus and IL-4 for alternative polarization. When assessing the macrophage surface marker CD11b, the total percentage of CD11b^+^ cells under M1 and M2 polarization conditions was similar while the MFI was slightly higher in M2-like macrophages independent of the usage of GM-CSF or M-CSF (Fig. 1A). Further, we observed high expression of CD14 on all cells under M1 and M2 polarizing conditions irrespective of GM-CSF or M-CSF differentiation with a higher CD14 expression in M1-like macrophages (Fig. 1B). For both classical macrophage markers CD68 and MHC class II molecules (Fig. 1C and D) we observed no differences in all four conditions tested. Similarly, on whole genome level GM-CSF and M-CSF induced M0 macrophages showed a very similar gene expression profile (Figure S1A–B). Of note, when the IL-4 signal was provided to monocytes from the beginning of the differentiation period, immature dendritic cells were generated showing a distinct transcriptome (Figure S2A–B) with a typical loss of macrophage markers such as CD14 or CD68 (Figure S2C).

When assessing markers previously associated with M1 or M2 polarization [28], a selective induction of the M1 marker CD64 in M1-like macrophages was observed in cultures differentiated with both GM-CSF and M-CSF (Fig. 1E) while CD86 only showed an M1-specific expression in GM-CSF differentiated cells (Fig. 1F). Assessment of these markers following other M1-associated polarization signals, e.g. LPS, TNF-α or combinations thereof resulted in comparable results (Figure S3). Inversely, strong induction of the M2-marker CD23 was observed in IL-4 polarized macrophages with significantly higher induction in GM-CSF polarized M2-like macrophages (Fig. 1G). As we were mainly interested in macrophage polarization under inflammatory conditions, we chose to differentiate monocytes with GM-CSF for 3 days for further experiments prior to polarization with either IFN-γ or IL-4 as model signals.

### Characterization of M1- and M2-like Macrophages by Microarray-based Gene Expression Profiling

Most recently it has been suggested that assessment of macrophage polarization in humans cannot solely rely on few cell surface markers but should be accommodated by gene expression profiling [16]. Using one of the most recent array generations, gene expression profiling was performed on unpolarized and polarized macrophages derived from seven healthy donors. To determine sample relationships, PCA (Fig. 2A) and hierarchical clustering (Fig. 2B) based on variable genes were performed and showed segregation of the samples by polarization state. Comparing our data with publically available datasets from M1- and M2-like macrophages generated with earlier array versions we observed concordant gene expression patterns (Figure S4) [3]. Heatmap visualization of known M1- and M2-like macrophage markers (Fig. 2C) further demonstrated that the genes encoding for the surface molecules FCGR1A and FCGR1B (both representing CD64) and CD86, the cytokine/chemokine genes CXCL10, CXCL9, IL-1B, IL-6, CXCL11, TNF, IL-23A, and the genes encoding for the intracellular protein GBP1 were increased in M1-like macrophages, results similar to what has been previously reported for M1 polarization [3], [29], [30]. Inversely, M2-associated genes encoding for the surface molecules FCER2 (CD23), IL27RA, and CLEC4A, the chemokine genes CCL22, CCL18, and CCL17, and the intracellular protein F13A1 were increased in the M2-like macrophages [29]–[31]. Using a fold-change difference of ≥2 and a p-value <0.05 to determine differently expressed genes between M1 and M0 and M2 and M0 macrophages respectively, we observed 757 M1 and 436 M2 specific genes (Figure 2D).

To further illustrate differential macrophage polarization, we generated a priori knowledge based M1-associated (Fig. 2E) and M2-associated (Fig. 2F) networks applying EGAN [25] using genes significantly upregulated in M1- (FC >2) respectively M2-polarized cells (FC >1.65). When applying expression values from the comparison of M2-like with M0 macrophages on the M1-associated network, the vast majority of genes showed either no change or even a reduction in expression, likely to represent an active repression of M1-associated genes in M2-like macrophages (Fig. 2E). Only few genes showed a simultaneous increase in expression, and these genes represented common cell cycle associated genes. Similarly, members of the M2-associated network were mostly not changed or even reduced in M1-like macrophages (Fig. 2F).

### Increase in Overall Transcriptome Information by RNA-seq

Gene expression profiling using microarrays has recently been suggested to be inferior to newer sequencing based technologies in providing genome-wide transcriptome information [14]. To address the potential information increase for macrophages, RNA-seq was performed on *in vitro* generated M1- and M2-like macrophages. After quality filtering, we obtained 15.0±2.8 million and 20.4±9.2 million reads for the M1- and M2-like macrophage cDNA libraries (Table 1). Consistent with RNA-seq data obtained from other eukaryotic cells [32] the majority of sequencing reads for M1- and M2-like macrophages mapped to annotated exons (Refseq transcripts). The remaining reads mapped to exon-intron boundaries, introns, or other uncharacterized intergenic regions (Table 1). RPKMs are measures of individual transcript abundance in RNA-seq datasets and have been shown to be highly accurate across multiple cell types [32]. We used CASAVA to assign RPKMs. To compare RNA-seq and microarray data we cross-annotated RNA-seq and microarray data using HGNC symbols. In human M1- and M2-like macrophages, 11317 and 11466 Refseq genes were expressed applying a previously defined optimal threshold (0.3 RPKM) for gene expression (Fig. 3A) [33]. The present call rate for Refseq genes for M1- (n = 10155) and M2-like macrophages (n = 10418) was only slightly lower when using microarrays (Fig. 3A). Furthermore, when assessing the levels of mRNA expression on a global scale a high correlation between RNA-seq and microarray data – similar to other cells [32] – was observed for M1- and M2-like macrophages (Fig. 3B).

### RNA-seq Reveals Differential Expression at Significantly Higher Resolution

RNA-seq showed a significantly increased dynamic range over background mainly due to significantly lower background levels. This suggested that the assessment of differential expression using RNA-seq might lead to an improved resolution in comparison to array-based data. Applying standard filter criteria (FC ≥1.5, p<0.05, RPKM ≥0.3) revealed a total of 1736 genes elevated in M1- versus M2-like macrophages by RNA-seq, while 834 genes were recognized by array analysis (Fig. 3C). Similarly, 822 genes were identified as being elevated in M2- versus M1-like macrophages by RNA-seq, while 786 genes were detected by array analysis (Fig. 3D). More importantly, when categorizing differentially expressed genes according to their level of differential expression, RNA-seq data clearly revealed up to 4-fold more genes with FC >4 for M1- and M2-associated genes (Fig. 3C and D), which was similarly true for M1-associated genes at lower levels (1.5< FC <4). To reveal potential reasons for this difference on the single-gene level we utilized FC-FC plotting, correlating RNA-seq and array-based data for individual genes (Fig. 3E). The majority of genes showed similar behavior in both RNA-seq and microarray experiments, albeit the relative differences were higher in RNA-seq data (Fig. 3E). Altogether, we observed a considerable increase in the dynamic range of fold-differences in RNA-seq data with differences spanning six orders of magnitude in contrast to only four orders of magnitude in the microarray data (Fig. 3E and Figure S5). In addition, there was a significant number of genes showing differential expression in RNA-seq data (e.g. DUOX1, DUOX2, TBX21, GBP7) but not in the array data suggesting that the array data are not informative for this class of genes. As anticipated, when using Venn diagrams with a defined cutoff (-2≥ FC ≥2, p<0.05, RPKM ≥0.3) for data presentation (Fig. 3F), both RNA-seq and array data revealed 595 genes to be differentially expressed, but RNA-seq revealed 900 additional genes of which 3 we validated on protein level (Figure S6). Surprisingly, 308 genes were classified as being differentially expressed by array analysis alone (Fig. 3F). When further assessing these genes, it became apparent that these genes show a similar trend in the RNA-seq data but these differences did not yet reach statistical significance (Fig. 3G). In contrast, genes only identified by RNA-seq, clearly showed no differences when assessed by array analysis (Fig. 3H). Visualization of typical marker genes as depicted for array data in Fig. 2C demonstrated a comparable differentiation of M1- and M2-like macrophages when assessed by RNA-seq (Fig. 3I).

### Exon Resolution Transcriptome Analysis of Known Macrophage Markers

Another advantage of RNA-seq is to resolve gene expression on the exon level (Fig. 4). For the macrophage related genes CD68 (Fig. 4A–D), CD64 (Fig. 4E–H) and CD23 (Fig. 4I–L), RNA-seq data were visualized for the genomic loci of the respective genes and compared with array, qPCR, and FACS data. For CD68, RNA-seq data revealed similarly high expression for M1 and M2 macrophages for all exons with little variance in expression levels between donors (Fig. 4A and Figure S7A). Slightly higher variance was observed for both microarray (Fig. 4B) and qPCR data (Fig. 4C) while protein levels showed equal expression in all samples analyzed (Fig. 4D). For CD64, RNA-seq revealed complete absence of expression for all exons in M2-like macrophages with high expression in M1-like macrophages (Fig. 4E and Figure S7B), which was similarly observed by other technologies (Fig. 4F–H). For CD23, protein data suggest significantly elevated expression on M2-like macrophages with low level expression on M1-like macrophages (Fig. 4L), a result which was also observed for RNA-seq data (Fig. 4I and Figure S7C) as well as array (Fig. 4J) and qPCR (Fig. 4K). Similar results were obtained for other marker genes (data not shown). Additionally, we were able to detect classical M1/M2-markers not accessible using microarrays (Figure S8), suggesting that high-resolution RNA-seq data are predestined for exploration of genes not detectable using microarrays, novel marker genes, as well as biological principles of macrophage polarization.

### RNA-seq Ameliorates Network-based Analysis in M1- and M2-like Macrophages

To understand if RNA-seq would also enhance the understanding of biological principles of macrophage polarization we applied network analysis based on a priori information assessing the information content of RNA-seq data in comparison to array data. Genes expressed at elevated levels in M1 RNA-seq data (FC >4) were used for network generation (Fig. 5A). This primary RNA-seq based M1 network was subsequently used to visualize array-based gene expression (Fig. 5B). When genes at a lower level of differential expression (FC >2) were included 73% of the network was revealed in the array data and central hubs of the network were also categorized as being highly (FC >4) differentially expressed. However, only RNA-seq data revealed two gene clusters of immunomodulating proteins highly enriched in the M1 network, namely apolipoproteins L (APOL) (Fig. 5A and Figure S9) and the leukocyte immunoglobulin-like receptor (LILR) family (Fig. 5A and Figure S10) [34]–[36]. As exemplified for LILRB1 and APOL3 both genes were clearly identified by RNA-seq, qRT-PCR, and flow cytometry respective western blotting (Fig. 5E and F) but not by microarray analysis (data not shown). Applying the RNA-seq data-based M2 network (Fig. 5C) to the array data (Fig. 5D) revealed only 54% elevated genes and major network hubs were not revealed at all. Taken together, RNA-seq data were clearly enriched for biological a priori information in both M1 and M2 polarization.

### Identification of Splice Variants and RNA Chimaera in Differentially Stimulated Human Macrophages

It has recently been suggested that cell differentiation can result in usage of alternative gene transcripts or isoform switching [21]. We applied Cufflinks and Cuffdiff to illuminate switches in dominant promoter usage, transcription start sites (TSS), and coding sequences (CDS) [21]. This analysis revealed 9 genes with alternative promoters (Table S3), 28 genes using alternative TSS (Table S4), and 20 genes with different CDS in M1- and M2-like macrophages (Table S5). We analyzed one of these genes in greater detail. For the gene encoding PDZ and LIM domain 7 (enigma) (PDLIM7) we observed a slight but significant increase in M1-like macrophages for the complete locus in RNA-seq data (Fig. 6A) while the probes on the microarray revealed no significant changes (Fig. 6B). Previous screening projects suggested three different CDS for PDLIM7. Applying Cufflinks and Cuffdiff to M1 and M2 RNA-seq data clearly revealed differential expression of individual CDS (Fig. 6C). While PDLIM7 v1 was mainly expressed by M1-like macrophages, M2-like macrophages mainly expressed PDLIM7 v2 while no difference was observed for PDLIM7 v4. We validated the usage of these variants by version-specific qPCR (Fig. 6D). Taken together, these new findings might open new avenues towards the role of alternative splicing in macrophages potentially linking alternative transcript usage with macrophage polarization.

### New Markers for M1- and M2-like Macrophages Identified by Combined Transcriptome Analysis

In light of the still limited number of cell surface markers clearly distinguishing human M1- from M2-like macrophages, we interrogated the genes of the human surfaceome [37] for differential expression between M1- and M2-like macrophages. By this approach 475 surface molecules were found to be differentially expressed (Fig. 7A). As visualized in Fig. 7B, the cell surface molecules CD120b, TLR2, and SLAMF7 showed preferential expression in M1-like macrophages, which was true irrespective of differentiation of macrophages by GM-CSF or M-CSF (Figure S11A). Several surface molecules, including CD1a, CD1b, CD93 and CD226 were significantly increased in expression in M2-like macrophages (Fig. 7C and Figure S11B). Taken together, screening higher-resolution transcriptome data established by RNA-seq allows for the identification of novel genes related to specific polarization programs in macrophages.

## Discussion

Because of the enormous plasticity of human macrophages, the classification of polarization states on the basis of few cell surface markers will remain a substantial challenge [16]. Here, we addressed how RNA-seq based high-resolution transcriptome data can be utilized to better understand the biology of macrophage polarization. We observed a significant increase in dynamic range in RNA-seq data resulting in a significantly higher number of genes determined to be significantly differentially expressed. This was true despite the fact that we used seven biological replicates for array analysis but only three samples for RNA-seq. A priori information based network analysis further supported that the increased information content of RNA-seq data uncovered novel aspects of macrophage biology, which was illustrated by the recognition of differential expression of numerous family members of two gene families, namely the apolipoprotein L family and leukocyte immunoglobulin-like receptors. APOLs constitute a new class of apolipoproteins expressed by macrophages as they serve as lytic factors against invading pathogens, e.g. African trypanosomes inducing programmed cell death as well as inhibiting intracellular infection by Leishmania [34], [35]. LILRs have been associated with balancing the effects of Toll-like receptor signaling, suggesting an important role of LILRs both in the initiation but also cessation of inflammatory responses mediated by macrophages [36]. Another aspect enhancing our knowledge about the polarization biology of macrophages was the identification of several genes with differential usage of alternative promoters and transcription start sites as well as differential splicing variants between M1- and M2-like macrophages. As visualized for PDLIM7, an intracellular scaffold protein that contains a PDZ domain and three LIM domains linked to mitogenic signaling through actin cytoskeleton organization [38], regulating Tbx5 transcriptional activity [39], and suppressing p53 activity [40], RNA-seq revealed significant differences in splice variant usage for M1- and M2-like macrophages potentially linking p53 regulation with macrophage polarization [41]. Usage of splice variant-specific qPCR reactions supported these findings while this differential regulation was not revealed by microarray analysis. Altogether we detected differential promoter usage, transcription start site usage and splice variant usage in over 50 gene loci, a number that was surprisingly low taking into account that such mechanisms of transcriptional regulation have been suggested for the majority of gene loci in mammalian genomes [42].

While studies in other cell systems suggested that RNA-seq data will further improve cell characterization [13]–[15], the direct assessment of the new technology in macrophage polarization was necessary to estimate its potential information gain. Both, increased dynamic range and the identification of transcripts that were missed by microarray analysis were major reasons for the discovery of novel genes associated with either M1- or M2-polarization. Nevertheless, despite a lower number of informative transcripts in the microarray data, 73% of the major M1-network was still revealed – at least when using transcripts defined to be enriched in M1-like macrophages. However, this rate dropped to only 54% in the M2-network and major hubs like MYC and TP53 where only revealed by RNA-seq data in M2-like macrophages. Overall these findings point towards an advantage of RNA-seq data, when the endpoint of the analysis is the identification of novel biological mechanisms.

An important aspect of genomic characterization is the identification of novel marker genes in macrophage polarization [16]. When focusing on genes being part of the human surfaceome in most cases RNA-seq data revealed larger differences between M1-like and M2-like cells when compared to microarray data. Nevertheless, some genes only reached significant differential expression in the array data clearly pointing toward the necessity to include a large enough number of biological replicates also when applying RNA-seq. On the other hand, a subset of genes showed the well-known background noise effect in the microarray data resulting in non-significant differences between the two cell types. Irrespective of these different shortcomings of the two technologies, the overall differences between the two techniques in this defined gene space were less obvious suggesting that both technologies are well suited for cell surface marker identification. Taken together, we introduced several new marker genes for which we established FACS assays that can be used to distinguish between M1 and M2 polarization of macrophages and that can be combined with the analysis of common macrophage markers.

The identification of novel marker genes distinguishing human M1-like and M2-like macrophages opens new avenues towards understanding the biology of differentially polarized macrophages. One of the M1-marker identified in this study, namely CD120b (TNFR2) has been linked to cell survival, activation and even proliferation in other cell types such as T cells [43]. In contrast to TNFR1, TNFR2 preferentially leads to NFκB activation. Whether this is true in myeloid cells as well requires further investigation. However, earlier studies already suggested that production of TNF-α in macrophages might be interpreted as a phenotype-stabilizing feed-forward loop [44] and TNFR2 might actually play an important role in such a process.

SLAMF7 was originally identified as a NK cell-associated surface molecule [45]. Subsequently, it was shown to be expressed on lymphocytes and monocytes [46]. More recently, a reduced expression on monocytes and NK cells with a simultaneous increase of SLAMF7 on B cells was observed in patients with lupus erythematosus [47]. The strongest link to SLAMF7 as an M1 marker gene comes from observations in intestine allograft rejection, demonstrating that tissue macrophages derived from patients rejecting the graft showed elevated levels of SLAMF7 [48]. It would be interesting to see if macrophages in other settings of transplant rejection are also enriched for this novel M1 marker gene. Considering the identification of single specific marker genes for macrophage polarization our findings clearly point to the necessity for multi-parameter analysis instead. This can be exemplified by the differential expression of CD1a and CD1b, two cell surface molecules that are mainly studied in context of antigen presentation by dendritic cells [49]. Previous reports suggested upregulation of CD1 proteins on human monocytes by GM-CSF [50]. However, we clearly present evidence that expression is induced in both M-CSF and GM-CSF driven macrophages and polarization towards M2-like macrophages is significantly increasing expression of CD1a and CD1b suggesting that they might be up-regulated on tissue macrophages in an M2-driving environment. This is similarly true for CD93, which was originally identified to be expressed on early hematopoietic stem cells and B cells [51]. CD93 is involved in biological processes such as adhesion, migration, and phagocytosis [52], [53]. CD93 expressed on myeloid cells can be shed from the cell surface and the soluble form seems to be involved in differentiation of monocytes towards a macrophage phenotype [54]. Since soluble CD93 has been implicated in inflammatory responses, it will be important to further elucidate how polarization-induced differential expression of CD93 contributes to specific inflammatory responses. Another surprising finding is the differential expression of CD226 between human M1- and M2-like macrophages, a molecule initially shown to be involved in cytolytic function of T cells [55]. Subsequently, it could be shown that CD226 has additional functions including the regulation of monocyte migration through endothelial junctions [56]. Similar to the other M2-associated markers, so far little is known about CD226 on polarized macrophages. Since CD226 expression levels on lymphocytes have been implicated in autoimmune diseases [57] further research is necessary to understand its role in the myeloid compartment during such processes.

Overall, by using RNA-seq we introduce a high-resolution transcriptome analysis of human macrophages unraveling novel insights into macrophage polarization. While previously established transcriptome datasets addressing macrophage biology are still very suitable to assess important biological and medical questions, a deeper understanding of transcriptional regulation during macrophage polarization will require higher resolution that is provided by current and future RNA-seq technologies. Moreover, the novel cell surface markers will help to better understand macrophage programs and functions in human disease.

## Supporting Information

Figure S1 **Purity of human monocytes and macrophages after isolation and during cultivation.** (A) Purity of isolated human monocytes was determined by CD14 antibody staining and subsequent flow cytometric analysis. (B) Purity of GM-CSF and M-CSF differentiated human macrophages on day 3 was determined by CD14 antibody staining and subsequent flow cytometric analysis. (C) Purity of M1-like and M2-like macrophages differentiated in the presence of GM-CSF and M-CSF on day 6 was determined by CD14 and CD11b antibody staining and subsequent flow cytometric analysis. Isotype controls shown as dotted lines. (A–C) Data are representative for all experiments, each with cells derived from a different donor.
(TIF)

Figure S2 **Characterization of human macrophages derived from CD14^+^ peripheral blood monocytes by M-CSF and GM-CSF differentiation.** Whole genome expression analysis was performed to determine similarity of M-CSF and GM-CSF differentiated macrophages. (A) Principle component analysis of human unpolarized macrophages differentiated with M-CSF (M-CSF MΦ) or GM-CSF (GM-CSF MΦ) as well as immature dendritic cells, B cells and T cells using all probes. (B) Gene expression in M-CSF versus GM-CSF differentiated macrophages as fold change versus fold change plot comparing M-CSF or GM-CSF differentiated macrophages with immature dendritic cells using probes with fold-changes ≤−1.5 or ≥1.5 in one of the comparisons. (C) Expression of CD14 and CD68 using whole genome expression analysis.
(TIF)

Figure S3 **Phenotypic characterization of human M1-like macrophages derived from CD14^+^ peripheral blood monocytes.** Expression of classical M1 markers after polarization of GM-CSF generated macrophages with IFN-γ, LPSu, TNF-α or IFN-γ and LPSu. Surface expression of lineage markers CD14 and CD11b as well as surface expression of the typical M1 markers CD86 and CD64 was assessed by flow cytometry.
(TIF)

Figure S4 **Comparison of gene expression data of M1-like and M2-like macrophages with a published dataset (Martinez et al., J Immunol 2006).** Comparison of gene expression of GM-CSF and M-CSF differentiated (A) M1-like and (B) M2-like macrophages. Shown are fold change versus fold change plots comparing GM-CSF differentiated (A) M1-like vs M0 macrophages and (B) M2-like vs M0 macrophages with M-CSF differentiated macrophages using all cross-annotated genes.
(TIF)

Figure S5 **Comparison of RNA-seq and microarray analysis.** Gene expression in M1- versus M2-like macrophages as fold change versus fold change plot comparing microarray analysis with RNA-seq using only Refseq genes differentially expressed in microarrays.
(TIF)

Figure S6 **Flow cytometric assessment of genes identified by RNA-seq.** (A) CD89, (B) CD82, and (C) CD200R protein expression in human M1- and M2-like macrophages. was determined by flow cytometry (left). *P<0.05 (Student’s t-test). Numbers in plots indicate mean fluorescence intensity. Data are representative of three independent experiments (mean and s.e.m.) each with cells derived from a different donor.
(TIF)

Figure S7 **Analysis of classical macrophage markers.** (A) CD68, (B), CD64, and (C) CD23 expression in human M1- and M2-like macrophages. Representative images of sequencing reads across genes expressed in human macrophages for all three donors analyzed. Pictures taken from the Integrative Genomics Viewer (IGV). The height of bars represents the relative accumulated number of 100-bp reads spanning a particular sequence. Gene maps (bottom portion of each panel, oriented 5′-3′ direction) are represented by thick (exons) and thin (introns) lines.
(TIF)

Figure S8 **Detection of classical macrophage genes by RNA-seq.** (A) IL-10 and (B) IL-18 expression in human M1- and M2-like macrophages. Left, expression as determined by microarray analysis using; middle, representative images of sequencing reads across genes expressed in human macrophages. Pictures taken from the Integrative Genomics Viewer (IGV). The height of bars represents the relative accumulated number of 100-bp reads spanning a particular sequence. Gene maps (bottom portion of each panel, oriented 5′-3′ direction) are represented by thick (exons) and thin (introns) lines. Right, relative mRNA expression by RNA-seq in M1- and M2-like macrophages. Data are representative of seven (microarrays, mean and s.d.) or three experiments (RNA-seq, mean and s.d.) each with cells derived from a different donor. *P<0.05 (Student’s t-test), n.s. = not significant.
(TIF)

Figure S9 **Analysis of the apolipoprotein L family genes in M1- and M2-like macrophages.** (A) APOL1, (B) APOL2, and (C) APOL6 expression in human M1- and M2-like macrophages. Left, relative expression as determined by RNA-seq; middle, representative images of sequencing reads across genes expressed in human macrophages. Pictures taken from the Integrative Genomics Viewer (IGV). The height of bars represents the relative accumulated number of 100-bp reads spanning a particular sequence. Gene maps (bottom portion of each panel, oriented 5′-3′ direction) are represented by thick (exons) and thin (introns) lines. Right, relative mRNA expression by qPCR in M1- and M2-like macrophages. Below, APOL1 protein expression as determined by immunoblotting. Data are representative of three experiments (RNA-seq, mean and s.d. and qPCR, mean and s.e.m.) and two experiments (immunoblotting, mean and s.e.m.) each with cells derived from a different donor. *P<0.05 (Student’s t-test).
(TIF)

Figure S10 **Analysis of the leukocyte immunoglobulin-like receptor family genes in M1- and M2-like macrophages.** (A) LILRA1, (B) LILRA2, (C) LILRA3, (D) LILRA5, and (E) LILRB3 expression in human M1- and M2-like macrophages. Left, relative expression as determined by RNA-seq; middle, representative images of sequencing reads across genes expressed in human macrophages. Pictures taken from the Integrative Genomics Viewer (IGV). The height of bars represents the relative accumulated number of 100-bp reads spanning a particular sequence. Gene maps (bottom portion of each panel, oriented 5′-3′ direction) are represented by thick (exons) and thin (introns) lines. Right, relative mRNA expression by qPCR in M1- and M2-like macrophages. (B) and (E), far right, protein expression as determined by flow cytometry. Data are representative of three experiments (RNA-seq, mean and s.d., qPCR, and flow cytometry, mean and s.e.m.) each with cells derived from a different donor. *P<0.05 (Student’s t-test).
(TIF)

Figure S11 **Identification of new macrophage polarization markers based on combined transcriptome analysis.** (A–B) Expression of novel M1- and M2-like macrophage markers on CD11b^+^CD14^+^ macrophages was determined by flow cytometry (left) of M1- and M2-like macrophages generated in the presence of M-CSF with quantification shown in the graph at the right. Expression of (A) CD120b, TLR2, and SLAM7 as well as (B) CD1a, CD1b, CD93, and CD226. *P<0.05 (Student’s t-test). Numbers in plots indicate mean fluorescence intensity. Data are representative of nine independent experiments (A, B; mean and s.e.m.) each with cells derived from a different donor.
(TIF)

Table S1 **qPCR oligonucleotides.**
(XLSX)

Table S2 **Isoform specific qPCR oligonucleotides.**
(XLSX)

Table S3 **Alternative promoter usage.**
(XLSX)

Table S4 **Alternative TSS usage.**
(XLSX)

Table S5 **Alternative CDS usage.**
(XLSX)

## References

[1] Geissmann F, Manz MG, Jung S, Sieweke MH, Merad M, et al (2010) Development of monocytes, macrophages, and dendritic cells. Science 327: 656–661. 20133564 10.1126/science.1178331 PMC2887389

[2] Waldo SW, Li Y, Buono C, Zhao B, Billings EM, et al (2008) Heterogeneity of human macrophages in culture and in atherosclerotic plaques. Am J Pathol 172: 1112–1126. 18321997 10.2353/ajpath.2008.070513 PMC2276432

[3] Martinez FO, Gordon S, Locati M, Mantovani A (2006) Transcriptional Profiling of the Human Monocyte-to-Macrophage Differentiation and Polarization: New Molecules and Patterns of Gene Expression. The Journal of Immunology 177: 7303–7311. 17082649 10.4049/jimmunol.177.10.7303

[4] Biswas SK, Mantovani A (2010) Macrophage plasticity and interaction with lymphocyte subsets: cancer as a paradigm. Nat Immunol 11: 889–896. 20856220 10.1038/ni.1937

[5] Mantovani A, Sica A (2010) Macrophages, innate immunity and cancer: balance, tolerance, and diversity. Curr Opin Immunol 22: 231–237. 20144856 10.1016/j.coi.2010.01.009

[6] Gustafsson C, Mjosberg J, Matussek A, Geffers R, Matthiesen L, et al (2008) Gene expression profiling of human decidual macrophages: evidence for immunosuppressive phenotype. PLoS One 3: e2078. 18446208 10.1371/journal.pone.0002078 PMC2323105

[7] Lehtonen A, Ahlfors H, Veckman V, Miettinen M, Lahesmaa R, et al (2007) Gene expression profiling during differentiation of human monocytes to macrophages or dendritic cells. J Leukoc Biol 82: 710–720. 17595377 10.1189/jlb.0307194

[8] Nau GJ, Richmond JF, Schlesinger A, Jennings EG, Lander ES, et al (2002) Human macrophage activation programs induced by bacterial pathogens. Proc Natl Acad Sci U S A 99: 1503–1508. 11805289 10.1073/pnas.022649799 PMC122220

[9] Lacey DC, Achuthan A, Fleetwood AJ, Dinh H, Roiniotis J, et al (2012) Defining GM-CSF- and Macrophage-CSF-Dependent Macrophage Responses by In Vitro Models. J Immunol 188: 5752–5765. 22547697 10.4049/jimmunol.1103426

[10] Heng TS, Painter MW (2008) Immunological Genome Project C (2008) The Immunological Genome Project: networks of gene expression in immune cells. Nat Immunol 9: 1091–1094. 18800157 10.1038/ni1008-1091

[11] Hashimoto S, Suzuki T, Dong HY, Yamazaki N, Matsushima K (1999) Serial analysis of gene expression in human monocytes and macrophages. Blood 94: 837–844. 10419873

[12] Lawrence T, Natoli G (2011) Transcriptional regulation of macrophage polarization: enabling diversity with identity. Nat Rev Immunol 11: 750–761. 22025054 10.1038/nri3088

[13] Ozsolak F, Milos PM (2011) RNA sequencing: advances, challenges and opportunities. Nature reviews Genetics 12: 87–98. 10.1038/nrg2934 PMC3031867 21191423

[14] Wang Z, Gerstein M, Snyder M (2009) RNA-Seq: a revolutionary tool for transcriptomics. Nature reviews Genetics 10: 57–63. 10.1038/nrg2484 PMC2949280 19015660

[15] Marioni JC, Mason CE, Mane SM, Stephens M, Gilad Y (2008) RNA-seq: an assessment of technical reproducibility and comparison with gene expression arrays. Genome Res 18: 1509–1517. 18550803 10.1101/gr.079558.108 PMC2527709

[16] Murray PJ, Wynn TA (2011) Protective and pathogenic functions of macrophage subsets. Nature reviews Immunology 11: 723–737. 10.1038/nri3073 PMC3422549 21997792

[17] R Development Core Team (2011) R: A Language and Environment for Statistical Computing.

[18] Gentleman RC, Carey VJ, Bates DM, Bolstad B, Dettling M, et al (2004) Bioconductor: open software development for computational biology and bioinformatics. Genome Biol 5: R80. 15461798 10.1186/gb-2004-5-10-r80 PMC545600

[19] Faria JC, Demetrio CGB (2010) bpca: Biplot of Multivariate Data Based on Principal Components Analysis.

[20] Fujita PA, Rhead B, Zweig AS, Hinrichs AS, Karolchik D, et al (2011) The UCSC Genome Browser database: update 2011. Nucleic Acids Res 39: D876–882. 20959295 10.1093/nar/gkq963 PMC3242726

[21] Trapnell C, Williams BA, Pertea G, Mortazavi A, Kwan G, et al (2010) Transcript assembly and quantification by RNA-Seq reveals unannotated transcripts and isoform switching during cell differentiation. Nature biotechnology 28: 511–515. 10.1038/nbt.1621 PMC3146043 20436464

[22] Langmead B, Trapnell C, Pop M, Salzberg SL (2009) Ultrafast and memory-efficient alignment of short DNA sequences to the human genome. Genome Biol 10: R25. 19261174 10.1186/gb-2009-10-3-r25 PMC2690996

[23] Trapnell C, Pachter L, Salzberg SL (2009) TopHat: discovering splice junctions with RNA-Seq. Bioinformatics 25: 1105–1111. 19289445 10.1093/bioinformatics/btp120 PMC2672628

[24] Trapnell C, Roberts A, Goff L, Pertea G, Kim D, et al (2012) Differential gene and transcript expression analysis of RNA-seq experiments with TopHat and Cufflinks. Nat Protoc 7: 562–578. 22383036 10.1038/nprot.2012.016 PMC3334321

[25] Paquette J, Tokuyasu T (2010) EGAN: exploratory gene association networks. Bioinformatics 26: 285–286. 19933825 10.1093/bioinformatics/btp656 PMC2804305

[26] Beyer M, Thabet Y, Muller RU, Sadlon T, Classen S, et al (2011) Repression of the genome organizer SATB1 in regulatory T cells is required for suppressive function and inhibition of effector differentiation. Nat Immunol 12: 898–907. 21841785 10.1038/ni.2084 PMC3669688

[27] Hamilton JA (2008) Colony-stimulating factors in inflammation and autoimmunity. Nat Rev Immunol 8: 533–544. 18551128 10.1038/nri2356

[28] Mantovani A, Sozzani S, Locati M, Allavena P, Sica A (2002) Macrophage polarization: tumor-associated macrophages as a paradigm for polarized M2 mononuclear phagocytes. Trends Immunol 23: 549–555. 12401408 10.1016/s1471-4906(02)02302-5

[29] Mantovani A, Sica A, Locati M (2005) Macrophage polarization comes of age. Immunity 23: 344–346. 16226499 10.1016/j.immuni.2005.10.001

[30] Mosser DM, Edwards JP (2008) Exploring the full spectrum of macrophage activation. Nat Rev Immunol 8: 958–969. 19029990 10.1038/nri2448 PMC2724991

[31] Wirnsberger G, Hebenstreit D, Posselt G, Horejs-Hoeck J, Duschl A (2006) IL-4 induces expression of TARC/CCL17 via two STAT6 binding sites. Eur J Immunol 36: 1882–1891. 16810739 10.1002/eji.200635972 PMC2988193

[32] Mortazavi A, Williams BA, McCue K, Schaeffer L, Wold B (2008) Mapping and quantifying mammalian transcriptomes by RNA-Seq. Nature methods 5: 621–628. 18516045 10.1038/nmeth.1226 PMC13303166

[33] Ramskold D, Wang ET, Burge CB, Sandberg R (2009) An abundance of ubiquitously expressed genes revealed by tissue transcriptome sequence data. PLoS computational biology 5: e1000598. 20011106 10.1371/journal.pcbi.1000598 PMC2781110

[34] Pays E, Vanhollebeke B (2009) Human innate immunity against African trypanosomes. Curr Opin Immunol 21: 493–498. 19559585 10.1016/j.coi.2009.05.024

[35] Samanovic M, Molina-Portela MP, Chessler AD, Burleigh BA, Raper J (2009) Trypanosome lytic factor, an antimicrobial high-density lipoprotein, ameliorates Leishmania infection. PLoS Pathog 5: e1000276. 19165337 10.1371/journal.ppat.1000276 PMC2622765

[36] Brown D, Trowsdale J, Allen R (2004) The LILR family: modulators of innate and adaptive immune pathways in health and disease. Tissue Antigens 64: 215–225. 15304001 10.1111/j.0001-2815.2004.00290.x

[37] da Cunha JP, Galante PA, de Souza JE, de Souza RF, Carvalho PM, et al (2009) Bioinformatics construction of the human cell surfaceome. Proceedings of the National Academy of Sciences of the United States of America 106: 16752–16757. 19805368 10.1073/pnas.0907939106 PMC2757864

[38] Nakagawa N, Hoshijima M, Oyasu M, Saito N, Tanizawa K, et al (2000) ENH, containing PDZ and LIM domains, heart/skeletal muscle-specific protein, associates with cytoskeletal proteins through the PDZ domain. Biochemical and biophysical research communications 272: 505–512. 10833443 10.1006/bbrc.2000.2787

[39] Camarata T, Krcmery J, Snyder D, Park S, Topczewski J, et al (2010) Pdlim7 (LMP4) regulation of Tbx5 specifies zebrafish heart atrio-ventricular boundary and valve formation. Developmental biology 337: 233–245. 19895804 10.1016/j.ydbio.2009.10.039 PMC2812577

[40] Jung CR, Lim JH, Choi Y, Kim DG, Kang KJ, et al (2010) Enigma negatively regulates p53 through MDM2 and promotes tumor cell survival in mice. The Journal of clinical investigation 120: 4493–4506. 21060154 10.1172/JCI42674 PMC2993588

[41] Matas D, Milyavsky M, Shats I, Nissim L, Goldfinger N, et al (2004) p53 is a regulator of macrophage differentiation. Cell death and differentiation 11: 458–467. 14713961 10.1038/sj.cdd.4401379

[42] Kapranov P, Willingham AT, Gingeras TR (2007) Genome-wide transcription and the implications for genomic organization. Nature reviews Genetics 8: 413–423. 10.1038/nrg2083 17486121

[43] Faustman D, Davis M (2010) TNF receptor 2 pathway: drug target for autoimmune diseases. Nat Rev Drug Discov 9: 482–493. 20489699 10.1038/nrd3030

[44] Popov A, Abdullah Z, Wickenhauser C, Saric T, Driesen J, et al (2006) Indoleamine 2,3-dioxygenase-expressing dendritic cells form suppurative granulomas following Listeria monocytogenes infection. The Journal of clinical investigation 116: 3160–3170. 17111046 10.1172/JCI28996 PMC1636691

[45] Boles KS, Stepp SE, Bennett M, Kumar V, Mathew PA (2001) 2B4 (CD244) and CS1: novel members of the CD2 subset of the immunoglobulin superfamily molecules expressed on natural killer cells and other leukocytes. Immunol Rev 181: 234–249. 11513145 10.1034/j.1600-065x.2001.1810120.x

[46] Murphy JJ, Hobby P, Vilarino-Varela J, Bishop B, Iordanidou P, et al (2002) A novel immunoglobulin superfamily receptor (19A) related to CD2 is expressed on activated lymphocytes and promotes homotypic B-cell adhesion. Biochem J 361: 431–436. 11802771 10.1042/0264-6021:3610431 PMC1222324

[47] Kim JR, Mathew SO, Patel RK, Pertusi RM, Mathew PA (2010) Altered expression of signalling lymphocyte activation molecule (SLAM) family receptors CS1 (CD319) and 2B4 (CD244) in patients with systemic lupus erythematosus. Clin Exp Immunol 160: 348–358. 20345977 10.1111/j.1365-2249.2010.04116.x PMC2883105

[48] Ashokkumar C, Ningappa M, Ranganathan S, Higgs BW, Sun Q, et al (2011) Increased expression of peripheral blood leukocyte genes implicate CD14+ tissue macrophages in cellular intestine allograft rejection. Am J Pathol 179: 1929–1938. 21854741 10.1016/j.ajpath.2011.06.040 PMC3181350

[49] Porcelli SA, Modlin RL (1999) The CD1 system: antigen-presenting molecules for T cell recognition of lipids and glycolipids. Annu Rev Immunol 17: 297–329. 10358761 10.1146/annurev.immunol.17.1.297

[50] Kasinrerk W, Baumruker T, Majdic O, Knapp W, Stockinger H (1993) CD1 molecule expression on human monocytes induced by granulocyte-macrophage colony-stimulating factor. J Immunol 150: 579–584. 7678276

[51] Greenlee-Wacker MC, Galvan M, Bohlson SS (2011) CD93: Recent Advances and Implications in Disease. Curr Drug Targets. 10.2174/138945012799424651 22206251

[52] McGreal EP, Ikewaki N, Akatsu H, Morgan BP, Gasque P (2002) Human C1qRp is identical with CD93 and the mNI-11 antigen but does not bind C1q. J Immunol 168: 5222–5232. 11994479 10.4049/jimmunol.168.10.5222

[53] Nepomuceno RR, Ruiz S, Park M, Tenner AJ (1999) C1qRP is a heavily O-glycosylated cell surface protein involved in the regulation of phagocytic activity. J Immunol 162: 3583–3589. 10092817

[54] Jeon JW, Jung JG, Shin EC, Choi HI, Kim HY, et al (2010) Soluble CD93 induces differentiation of monocytes and enhances TLR responses. J Immunol 185: 4921–4927. 20861352 10.4049/jimmunol.0904011

[55] Shibuya A, Campbell D, Hannum C, Yssel H, Franz-Bacon K, et al (1996) DNAM-1, a novel adhesion molecule involved in the cytolytic function of T lymphocytes. Immunity 4: 573–581. 8673704 10.1016/s1074-7613(00)70060-4

[56] Reymond N, Imbert AM, Devilard E, Fabre S, Chabannon C, et al (2004) DNAM-1 and PVR regulate monocyte migration through endothelial junctions. J Exp Med 199: 1331–1341. 15136589 10.1084/jem.20032206 PMC2211807

[57] Sinha S, Miller LM, Subramanian S, Burrows GG, Vandenbark AA, et al (2011) RTL551 treatment of EAE reduces CD226 and T-bet+ CD4 T cells in periphery and prevents infiltration of T-bet+ IL-17, IFN-gamma producing T cells into CNS. PLoS One 6: e21868. 21750737 10.1371/journal.pone.0021868 PMC3130056

