# Supplementary figures and images for: High-Resolution Transcriptome of Human Macrophages
Source: PLoS One. 2012 Sep 21;7(9):e45466. doi: 10.1371/journal.pone.0045466 (PMC3448669; doi:10.1371/journal.pone.0045466)

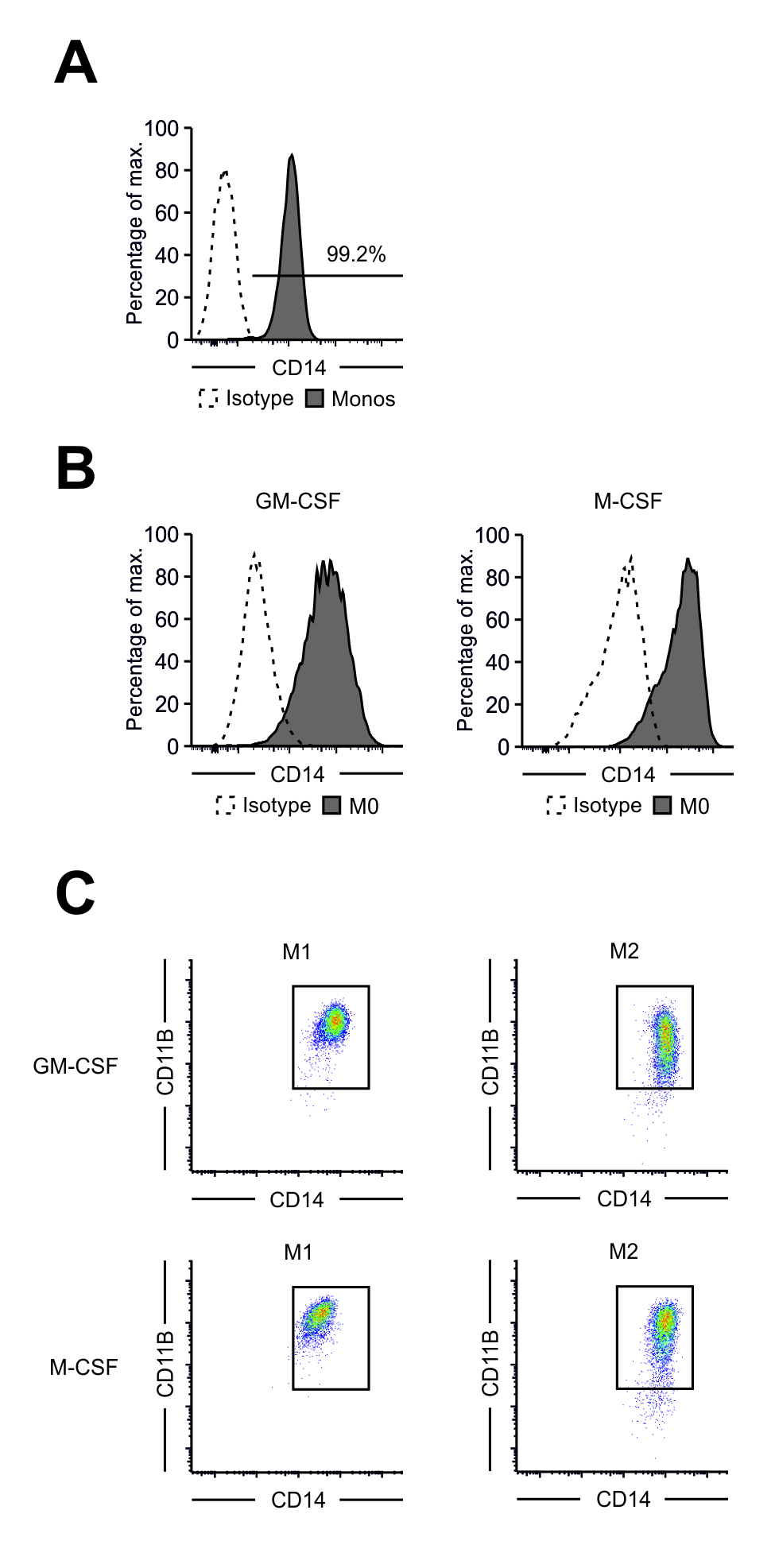

Supplement: Figure S1 — Purity of human monocytes and macrophages after isolation and during cultivation. (A) Purity of isolated human monocytes was determined by CD14 antibody staining and subsequent flow cytometric analysis. (B) Purity of GM-CSF and M-CSF differentiated human macrophages on day 3 was determined by CD14 antibody staining and subsequent flow cytometric analysis. (C) Purity of M1-like and M2-like macrophages differentiated in the presence of GM-CSF and M-CSF on day 6 was determined by CD14 and CD11b antibody staining and subsequent flow cytometric analysis. Isotype controls shown as dotted lines. (A–C) Data are representative for all experiments, each with cells derived from a different donor. (TIF) [file pone.0045466.s001.tif]

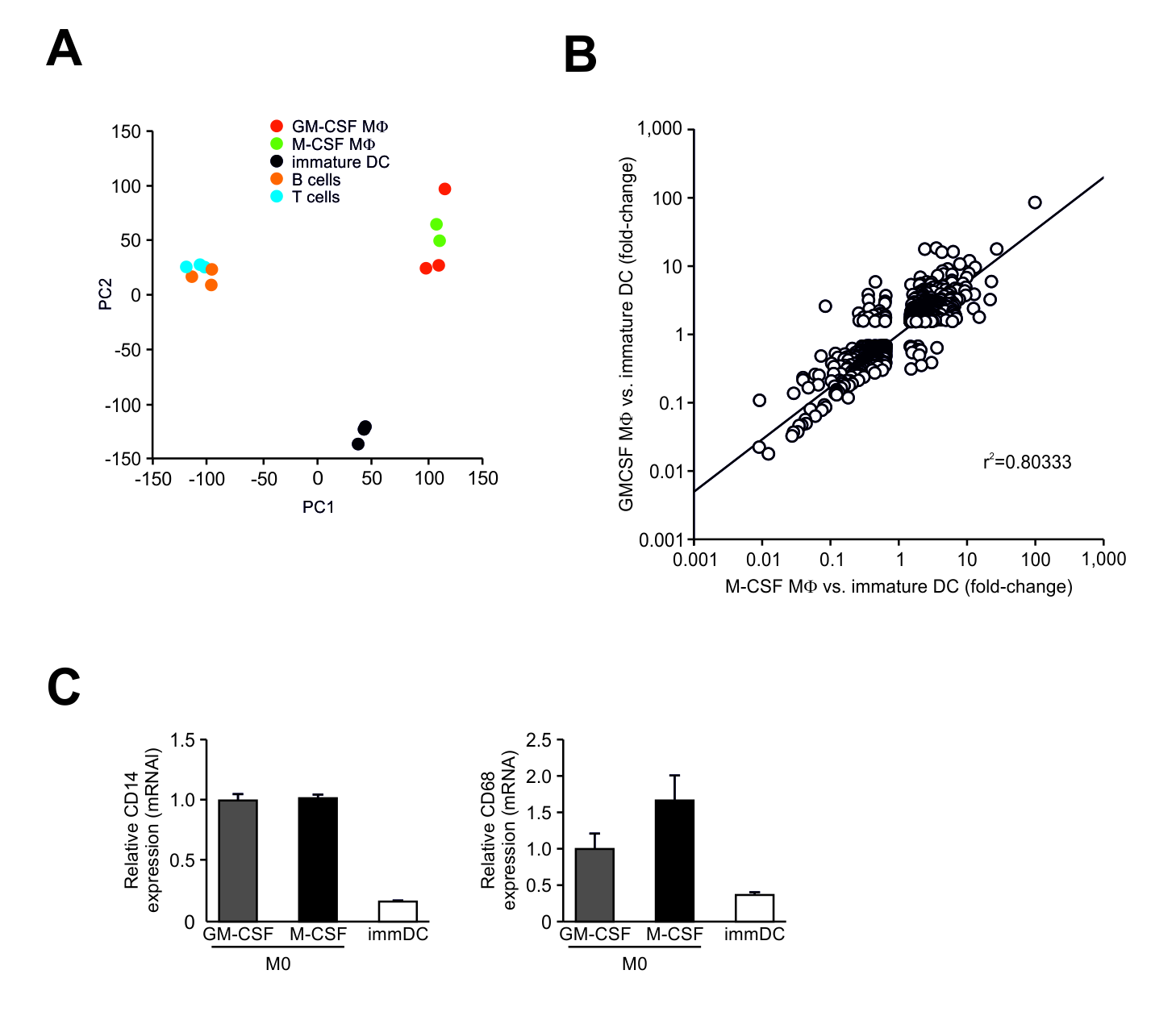

Supplement: Figure S2 — Characterization of human macrophages derived from CD14+ peripheral blood monocytes by M-CSF and GM-CSF differentiation. Whole genome expression analysis was performed to determine similarity of M-CSF and GM-CSF differentiated macrophages. (A) Principle component analysis of human unpolarized macrophages differentiated with M-CSF (M-CSF MΦ) or GM-CSF (GM-CSF MΦ) as well as immature dendritic cells, B cells and T cells using all probes. (B) Gene expression in M-CSF versus GM-CSF differentiated macrophages as fold change versus fold change plot comparing M-CSF or GM-CSF differentiated macrophages with immature dendritic cells using probes with fold-changes ≤−1.5 or ≥1.5 in one of the comparisons. (C) Expression of CD14 and CD68 using whole genome expression analysis. (TIF) [file pone.0045466.s002.tif]

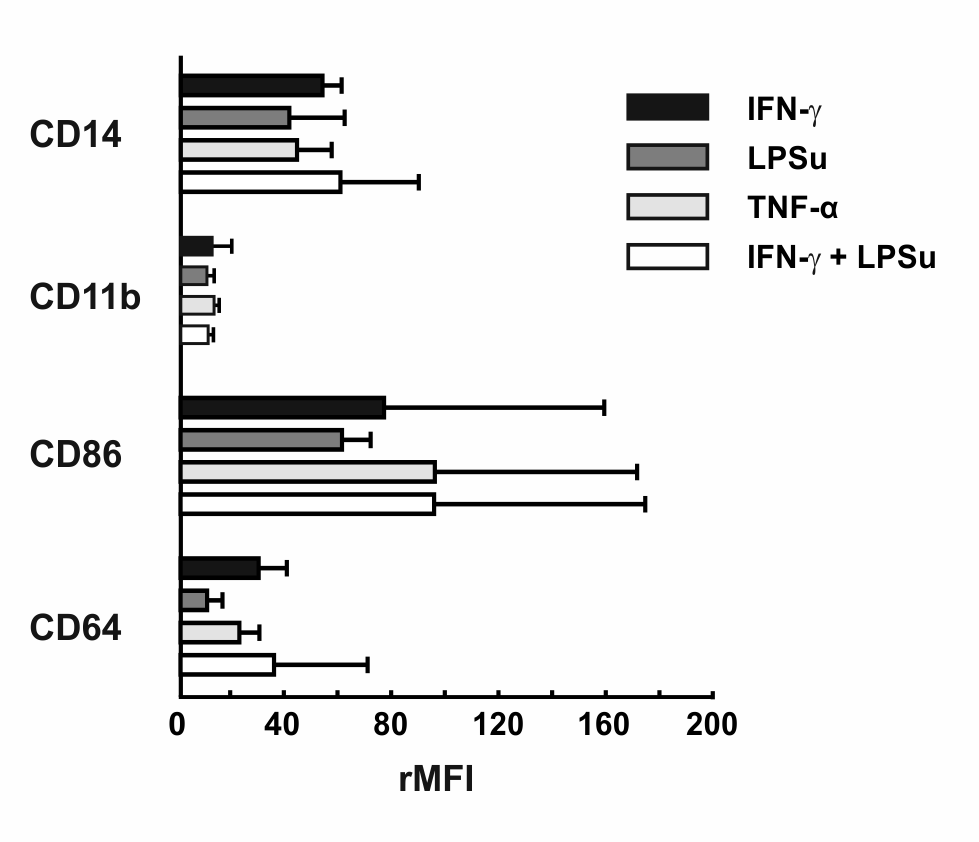

Supplement: Figure S3 — Phenotypic characterization of human M1-like macrophages derived from CD14+ peripheral blood monocytes. Expression of classical M1 markers after polarization of GM-CSF generated macrophages with IFN-γ, LPSu, TNF-α or IFN-γ and LPSu. Surface expression of lineage markers CD14 and CD11b as well as surface expression of the typical M1 markers CD86 and CD64 was assessed by flow cytometry. (TIF) [file pone.0045466.s003.tif]

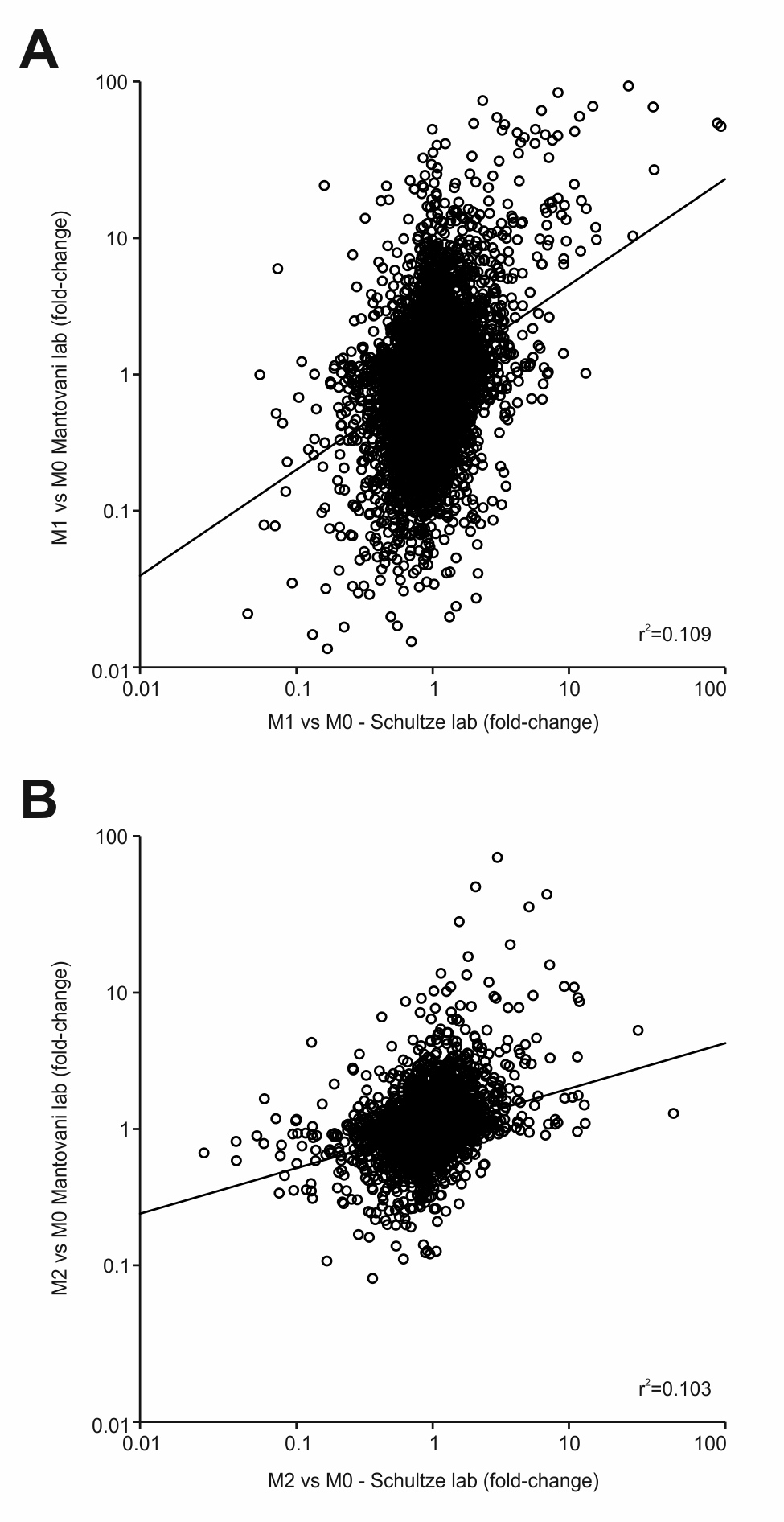

Supplement: Figure S4 — Comparison of gene expression data of M1-like and M2-like macrophages with a published dataset (Martinez et al., J Immunol 2006). Comparison of gene expression of GM-CSF and M-CSF differentiated (A) M1-like and (B) M2-like macrophages. Shown are fold change versus fold change plots comparing GM-CSF differentiated (A) M1-like vs M0 macrophages and (B) M2-like vs M0 macrophages with M-CSF differentiated macrophages using all cross-annotated genes. (TIF) [file pone.0045466.s004.tif]

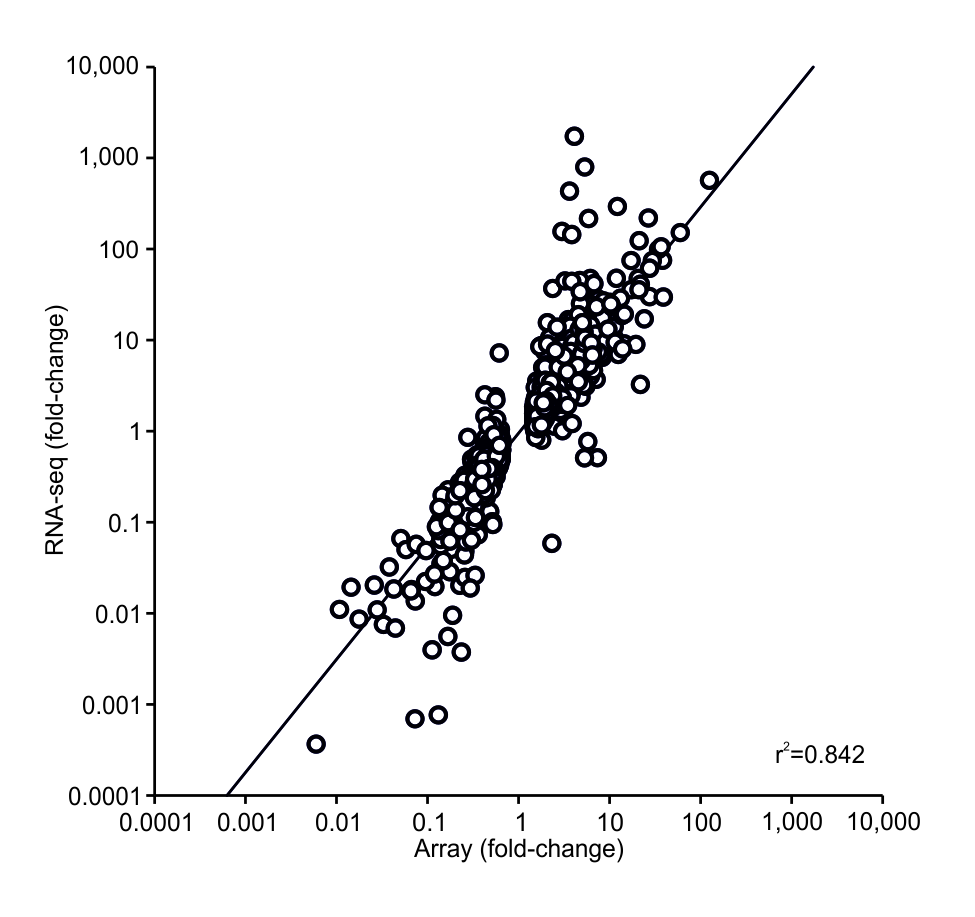

Supplement: Figure S5 — Comparison of RNA-seq and microarray analysis. Gene expression in M1- versus M2-like macrophages as fold change versus fold change plot comparing microarray analysis with RNA-seq using only Refseq genes differentially expressed in microarrays. (TIF) [file pone.0045466.s005.tif]

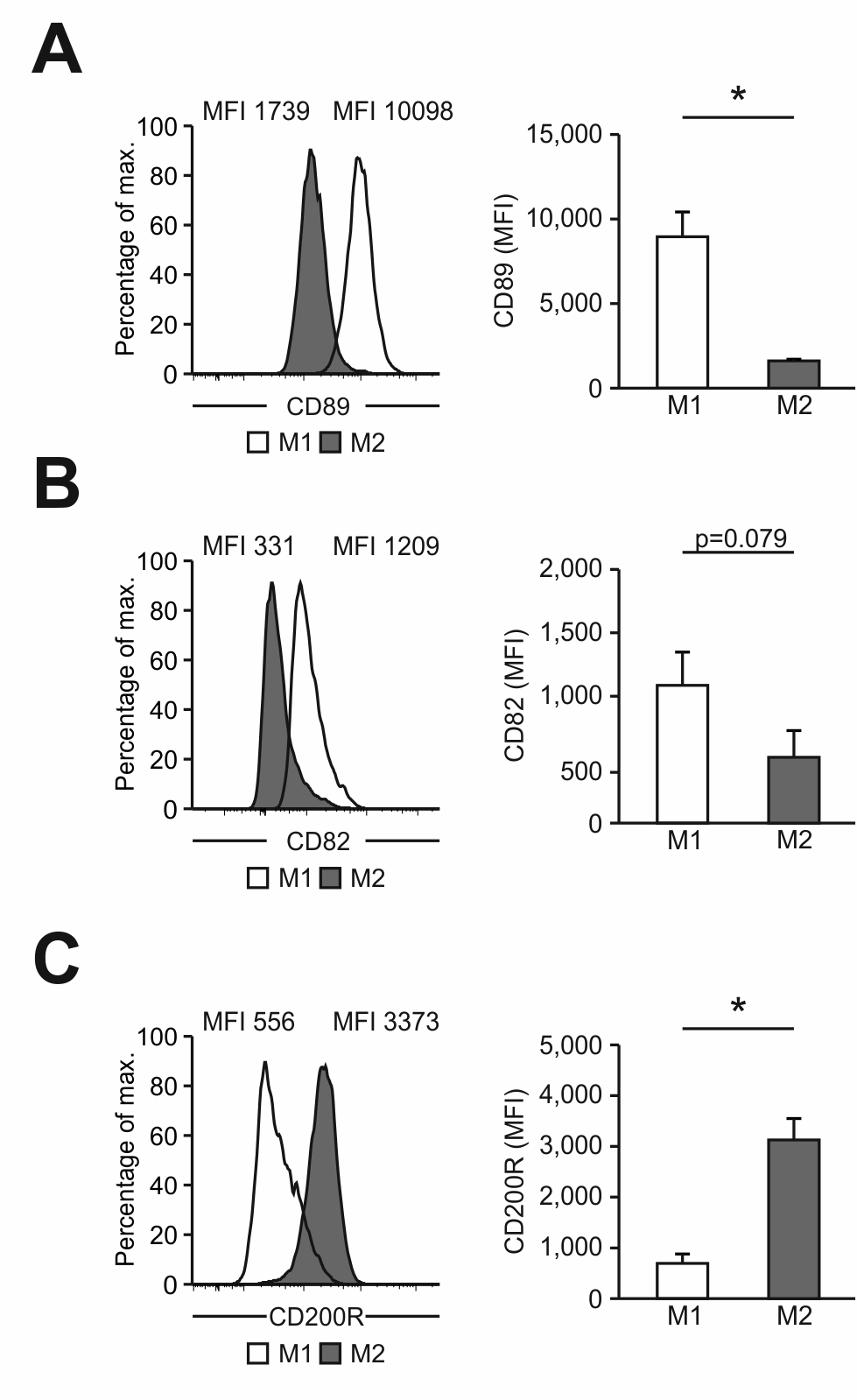

Supplement: Figure S6 — Flow cytometric assessment of genes identified by RNA-seq. (A) CD89, (B) CD82, and (C) CD200R protein expression in human M1- and M2-like macrophages. was determined by flow cytometry (left). *P<0.05 (Student’s t-test). Numbers in plots indicate mean fluorescence intensity. Data are representative of three independent experiments (mean and s.e.m.) each with cells derived from a different donor. (TIF) [file pone.0045466.s006.tif]

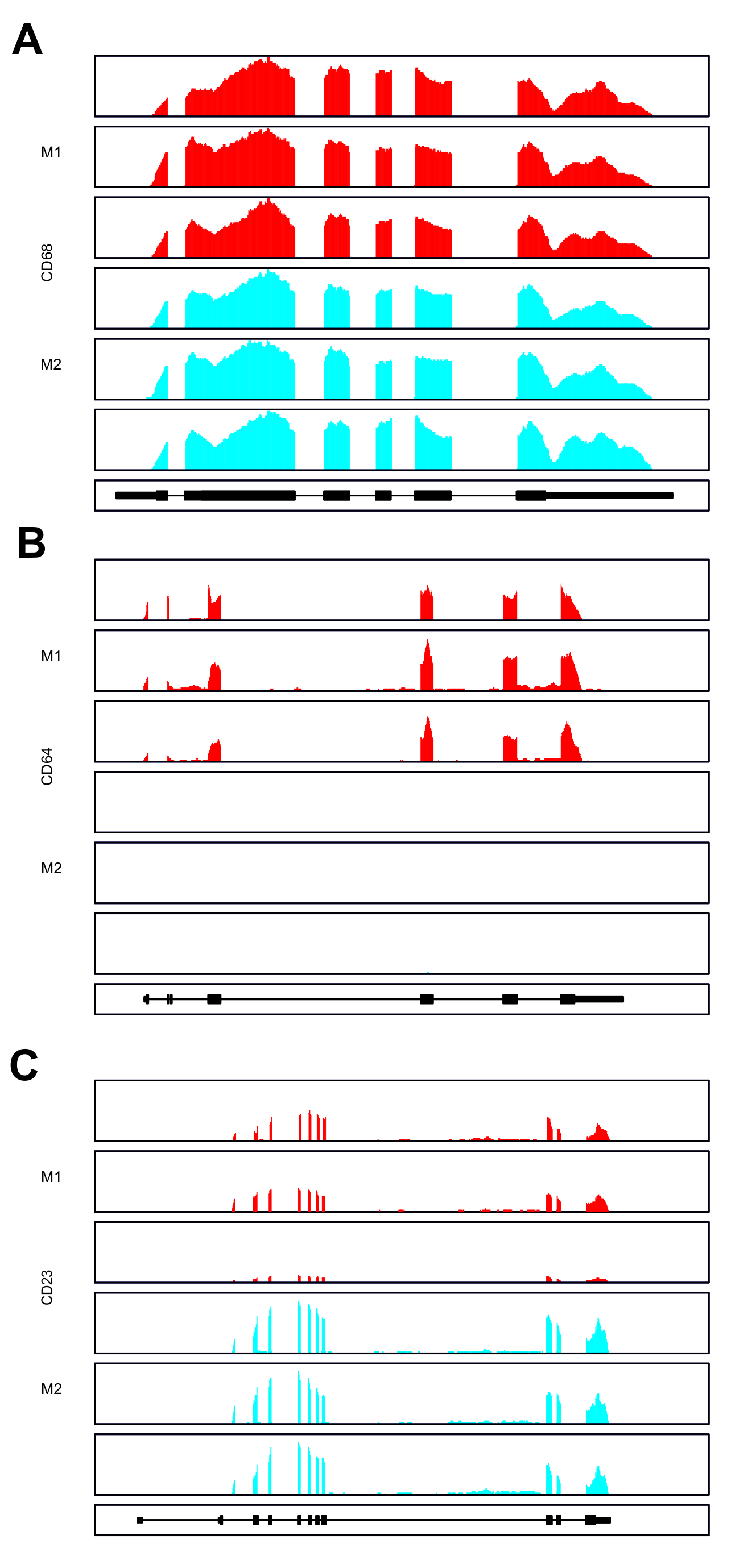

Supplement: Figure S7 — Analysis of classical macrophage markers. (A) CD68, (B), CD64, and (C) CD23 expression in human M1- and M2-like macrophages. Representative images of sequencing reads across genes expressed in human macrophages for all three donors analyzed. Pictures taken from the Integrative Genomics Viewer (IGV). The height of bars represents the relative accumulated number of 100-bp reads spanning a particular sequence. Gene maps (bottom portion of each panel, oriented 5′-3′ direction) are represented by thick (exons) and thin (introns) lines. (TIF) [file pone.0045466.s007.tif]

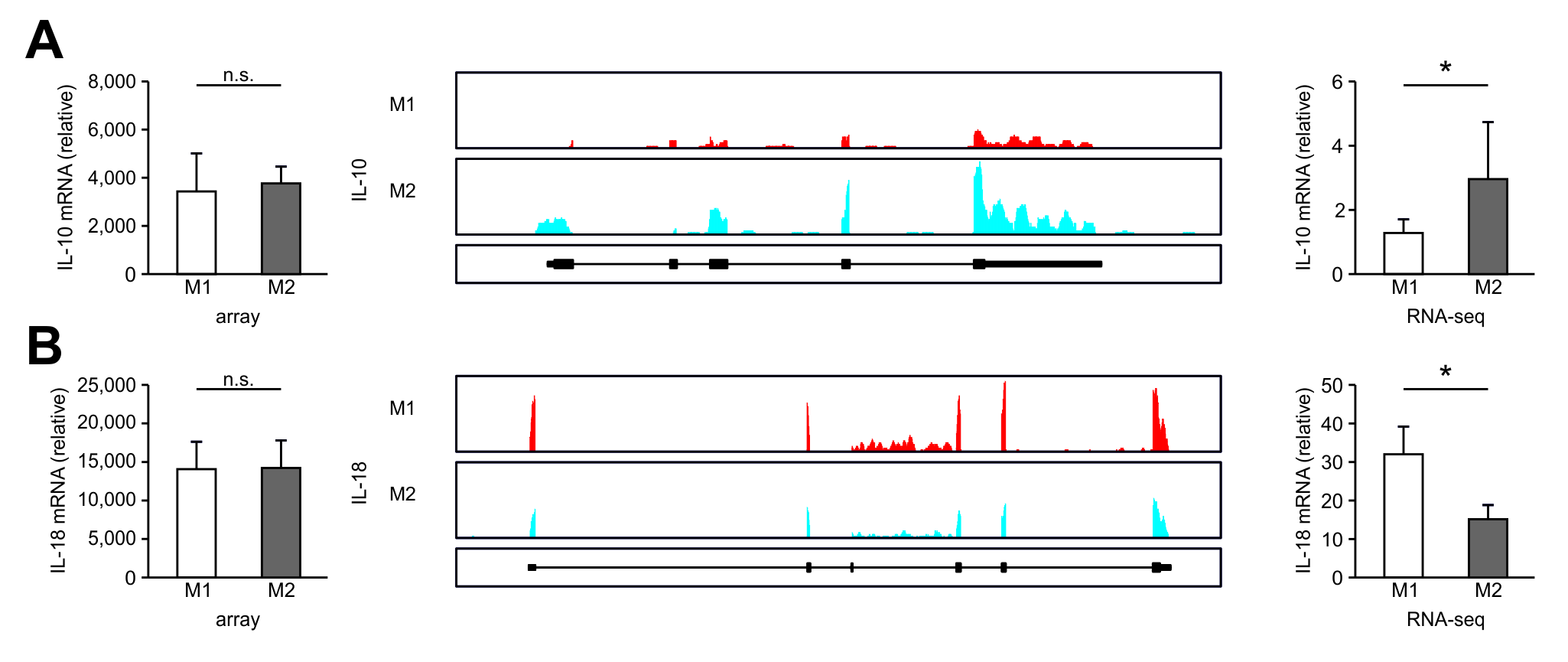

Supplement: Figure S8 — Detection of classical macrophage genes by RNA-seq. (A) IL-10 and (B) IL-18 expression in human M1- and M2-like macrophages. Left, expression as determined by microarray analysis using; middle, representative images of sequencing reads across genes expressed in human macrophages. Pictures taken from the Integrative Genomics Viewer (IGV). The height of bars represents the relative accumulated number of 100-bp reads spanning a particular sequence. Gene maps (bottom portion of each panel, oriented 5′-3′ direction) are represented by thick (exons) and thin (introns) lines. Right, relative mRNA expression by RNA-seq in M1- and M2-like macrophages. Data are representative of seven (microarrays, mean and s.d.) or three experiments (RNA-seq, mean and s.d.) each with cells derived from a different donor. *P<0.05 (Student’s t-test), n.s. = not significant. (TIF) [file pone.0045466.s008.tif]

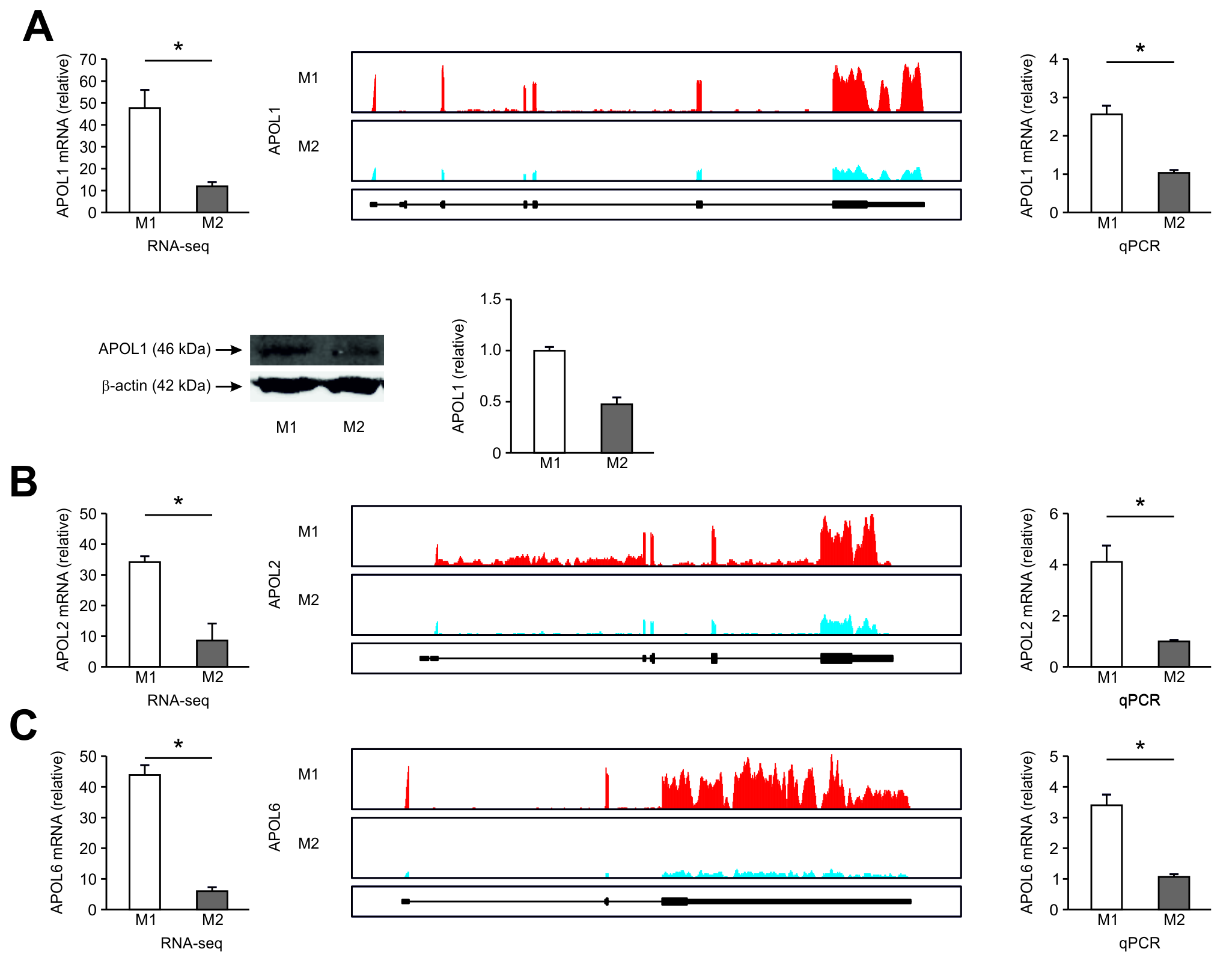

Supplement: Figure S9 — Analysis of the apolipoprotein L family genes in M1- and M2-like macrophages. (A) APOL1, (B) APOL2, and (C) APOL6 expression in human M1- and M2-like macrophages. Left, relative expression as determined by RNA-seq; middle, representative images of sequencing reads across genes expressed in human macrophages. Pictures taken from the Integrative Genomics Viewer (IGV). The height of bars represents the relative accumulated number of 100-bp reads spanning a particular sequence. Gene maps (bottom portion of each panel, oriented 5′-3′ direction) are represented by thick (exons) and thin (introns) lines. Right, relative mRNA expression by qPCR in M1- and M2-like macrophages. Below, APOL1 protein expression as determined by immunoblotting. Data are representative of three experiments (RNA-seq, mean and s.d. and qPCR, mean and s.e.m.) and two experiments (immunoblotting, mean and s.e.m.) each with cells derived from a different donor. *P<0.05 (Student’s t-test). (TIF) [file pone.0045466.s009.tif]

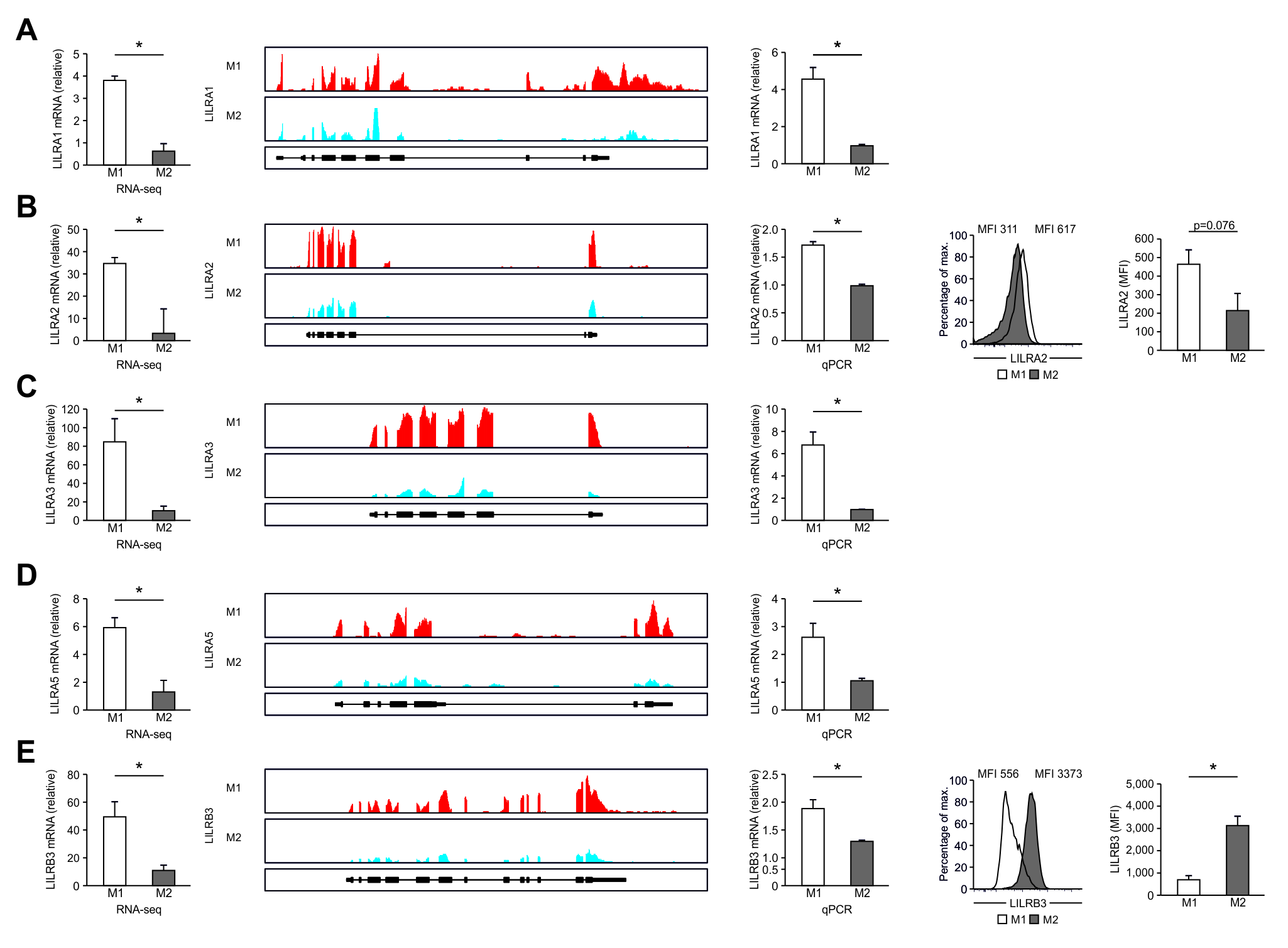

Supplement: Figure S10 — Analysis of the leukocyte immunoglobulin-like receptor family genes in M1- and M2-like macrophages. (A) LILRA1, (B) LILRA2, (C) LILRA3, (D) LILRA5, and (E) LILRB3 expression in human M1- and M2-like macrophages. Left, relative expression as determined by RNA-seq; middle, representative images of sequencing reads across genes expressed in human macrophages. Pictures taken from the Integrative Genomics Viewer (IGV). The height of bars represents the relative accumulated number of 100-bp reads spanning a particular sequence. Gene maps (bottom portion of each panel, oriented 5′-3′ direction) are represented by thick (exons) and thin (introns) lines. Right, relative mRNA expression by qPCR in M1- and M2-like macrophages. (B) and (E), far right, protein expression as determined by flow cytometry. Data are representative of three experiments (RNA-seq, mean and s.d., qPCR, and flow cytometry, mean and s.e.m.) each with cells derived from a different donor. *P<0.05 (Student’s t-test). (TIF) [file pone.0045466.s010.tif]

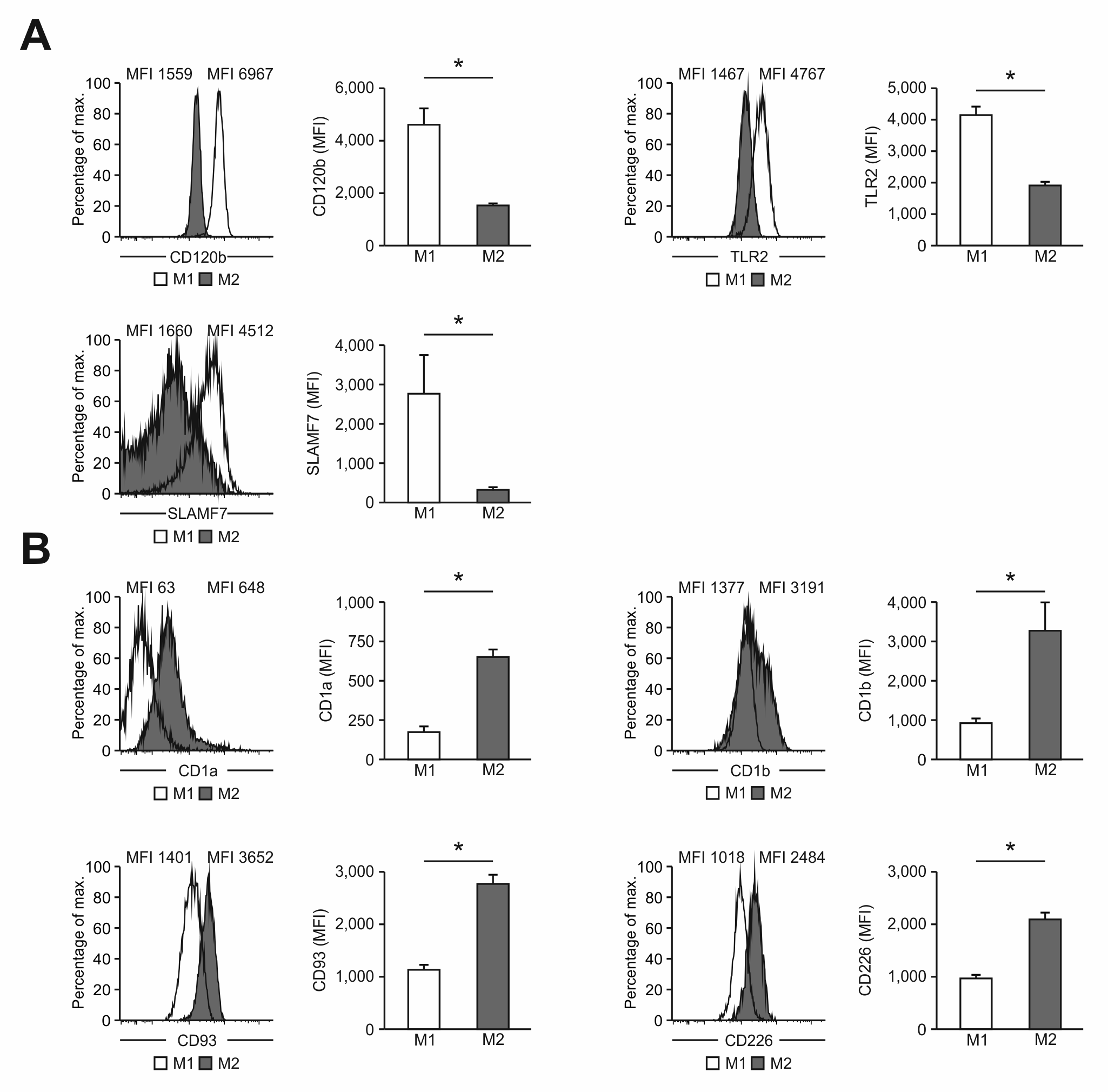

Supplement: Figure S11 — Identification of new macrophage polarization markers based on combined transcriptome analysis. (A–B) Expression of novel M1- and M2-like macrophage markers on CD11b+CD14+ macrophages was determined by flow cytometry (left) of M1- and M2-like macrophages generated in the presence of M-CSF with quantification shown in the graph at the right. Expression of (A) CD120b, TLR2, and SLAM7 as well as (B) CD1a, CD1b, CD93, and CD226. *P<0.05 (Student’s t-test). Numbers in plots indicate mean fluorescence intensity. Data are representative of nine independent experiments (A, B; mean and s.e.m.) each with cells derived from a different donor. (TIF) [file pone.0045466.s011.tif]
